# Polyphenol–Mediated Peptide Assembly Modulates Melittin Toxicity: A Structure–Activity Strategy for Neutralizing the Interface Affinity of Pore–Forming Toxins to Cell Membranes

**DOI:** 10.1021/acsami.5c09472

**Published:** 2025-08-25

**Authors:** Hojin Han, Julia C. Palchak, Johnathan R. Pinc, Michael Nguyen, Keira A. Atchley, Donald J. Darrell, Elaine S. Kim, Patrick H. Lee, Mary Claire Schleck, Alyssa R. Cornell, Joseph E. Buselmeier, Kate M. Bacon, Kaitlyn Zang, Nathan Burpeau, Justus M. Gabriel, Carol A. Anderson, F. John Burpo, Lucas B. Fallot, Simuck F. Yuk, Ryan Limbocker

**Affiliations:** † Department of Chemical and Biological Science and Engineering, 8531 United States Military Academy, West Point, New York 10996, United States; ‡ Photonics Research Center, United States Military Academy, West Point, New York 10996, United States

**Keywords:** biotoxin neutralization, membrane interfaces, membrane–toxin interactions, pore-forming agents, structure−activity relationship

## Abstract

Pore-forming agents can bind at the interface of and permeabilize cell membranes. Understanding and mitigating this mechanism is pragmatic for developing bionanomaterials and strategies against biologically active species that target the cell membrane. Herein, we explore the molecular interactions between melittin, a membrane-active pore-forming peptide from honeybee venom, and a series of structurally similar polyphenols. We sought to better understand the biophysical bases by which pore-forming toxins interact with cell membranes and to establish a materials-based strategy using small molecules to control peptide assembly and biotoxin activity at the membrane interface. Building on our previous discovery that epigallocatechin gallate reduces the membrane affinity of melittin by decreasing the extent of its solvent-exposed hydrophobicity and promoting its oligomerization into larger species that interact with a markedly lower affinity to cell membranes, we now establish a structure–activity relationship using five polyphenols. Combining biophysical measurements, assays using SH-SY5Y cells, and first-principles computational modeling, we show that the polyphenol-induced oligomerization of melittin correlates strongly with its reduced toxicity. Specifically, the degree of neutralization is predicted well by the binding affinity of the polyphenol to melittin and the resulting size of the supramolecular melittin-polyphenol complex, with larger assemblies exhibiting markedly diminished cytotoxicity due to the sequestration of the toxic, monomeric form of melittin. The stabilized melittin-polyphenol complexes also demonstrate differential resistances to dissociation using a chaotropic agent. These findings highlight the relevance of physicochemical properties in the ability of proteinaceous toxins to interface with cell membranes and suggest that modulating peptide assembly through molecular binding is a viable strategy to rationally assemble and control pore-forming toxins. This work offers a mechanistic framework for designing small molecule-stabilized biomaterials that can regulate interfaces, with relevance to nanomaterials and nanomedicine.

## Graphical abstract

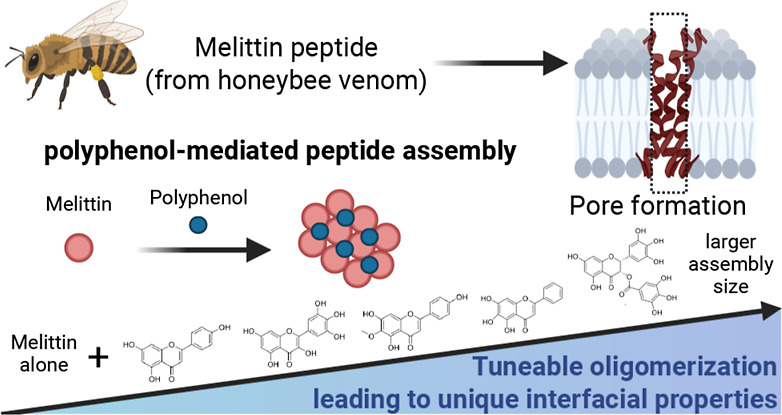

## Introduction

A range of pore-forming toxins are proteins that can cause the creation of transmembrane pores through diverse mechanisms.^1–4^ These proteins often undergo a transition from their monomeric form to a membrane-active, pore-forming conformation or complex.^3,5^ Melittin from honeybee venom is a 26-residue amphipathic, cationic peptide known to interact with and disrupt cellular membranes.^5,6^ Its sequence features two distinct regions, a hydrophobic N-terminal domain and a cationic, hydrophilic C-terminal region, and this peptide has a charge of +5 at neutral pH.^7,8^ When melittin binds to lipid bilayers, it can adopt an amphipathic α-helical conformation (Figure 1a).^7^ Specifically, melittin can bind within milliseconds to lipid membranes and adopt its amphipathic α-helical conformation, in either a perpendicular orientation that embeds in the membrane and is necessary to form a transmembrane pore, or it can bind parallel to the membrane in an inactive conformation.^8^ The inner diameter of the melittin pore has been measured at ∼4.4 nm in palmitoyl-2-oleoyl-*sn*-glycero-3-phosphocholine and dilauroyl phosphatidylcholine bilayers.^9^ A driving force for the binding of melittin to cell membranes is the electrostatic attraction between its positively charged residues and the negatively charged phospholipid heads of the bilayer.^10^ This interaction enhances its integration into the membrane, particularly in anionic lipid environments, where its α-helical structure further contributes to the destabilization of the membrane. As melittin inserts itself into the bilayer, it can disrupt membrane function and increase its permeability.^11–14^

**Figure 1:**
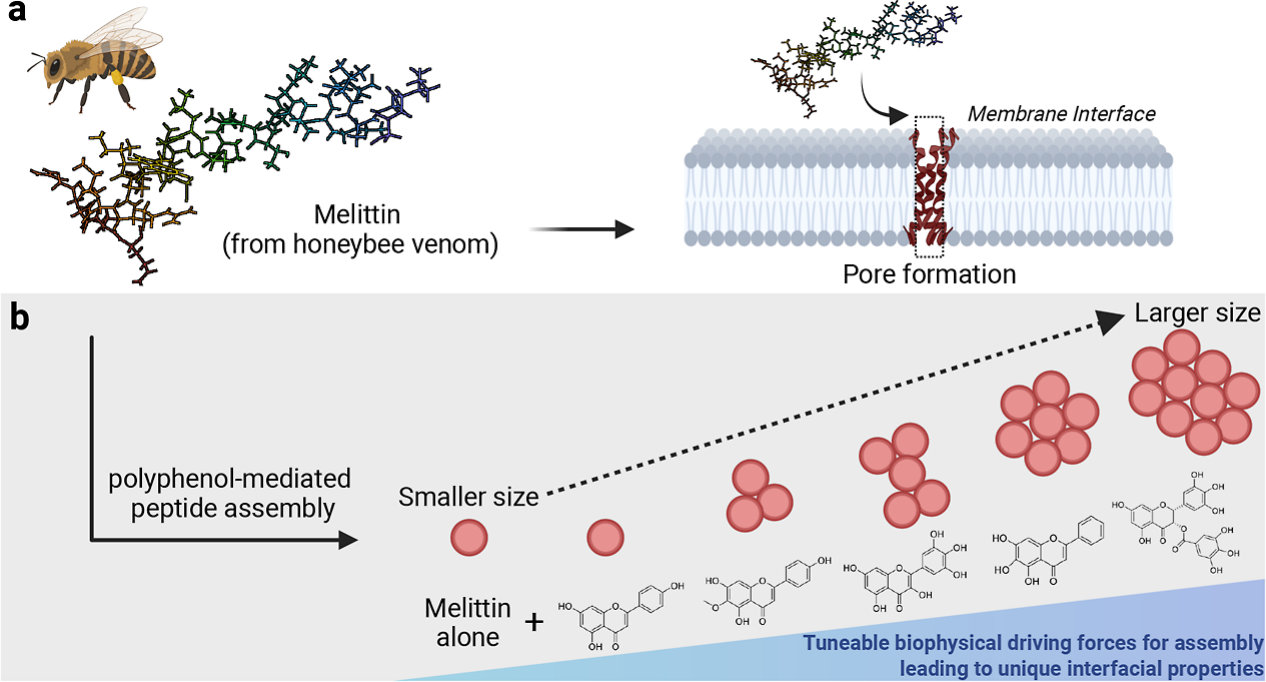
Polyphenol-mediated assembly of melittin into species with unique interfacial properties and pore-forming capacities. (a) Melittin from honeybee venom can adopt an α-helical secondary structure upon its interaction with the membrane interface in its ability to form transmembrane pores, as shown (PBD: 6DST). L.M. Ramirez, J. Pande, A. Shekhtman, Recombinant melittin (2019), 10.2210/pdb6DST/pdb. Melittin structure adapted from ref 5. Copyright 2018 American Chemical Society. (b) Polyphenols are a pragmatic scaffold to control with specificity the assembly of melittin into larger species with unique interfacial properties and pore-forming capacities. Created with Biorender.com.

The membrane interface is critical in maintaining cellular homeostasis by separating the intracellular and extracellular environments. Pore-forming agents can dramatically interact with and disrupt membranes, resulting in ion dyshomeostasis and cell death.^14,15^ Moreover, strategies are needed to capture proteins and potential biotoxins for a variety of nanomaterials and nanomedicine applications,^16^ such as biodefense,^17–19^ biocompatibility,^20–24^ decontamination,^25,26^ and encapsulation.^27^ Prior studies have shown that the physicochemical properties of melittin are associated with its interaction with the plasma membrane to form transmembrane pores.^14^ Our past work rationalized the pharmacological regulation of the membrane affinity of melittin, where the polyphenol epigallocatechin gallate (EGCG) directly interacted with this pore-forming peptide and prevented its interactions with membranes by inducing its oligomerization into large proteinaceous species, wherein melittin was highly sequestered.^14^

Polyphenols are natural products abundant in many plant species and are well-known for their antioxidant and neuroprotective properties.^28,29^ Structurally, polyphenols contain multiple aromatic rings with varying degrees of hydroxylation. They also can possess unique functional groups, and can adopt different three-dimensional structures depending on the central molecular scaffold. In this study, we sought to rationalize the structure–toxicity relationship and quantify the biophysical properties that regulate the binding affinity of melittin to membranes. To achieve this, we studied five structurally similar polyphenols: EGCG, apigenin, hispidulin, myricetin, and baicalein (Figure 1b, larger chemical structures shown in Figure S1). These represent a diverse group of polyphenols characterized by variations in the hydroxylation of the C2 phenyl group and the main backbone, resulting in varying degrees of hydrophobicity or hydrophilicity, with baicalein and apigenin being the most nonpolar (Log *P* = 1.90), followed by hispidulin (Log *P* = 1.78), EGCG (Log *P* = 0.92), and the most polar being myricetin (Log *P* = −0.04).^30^

Our results reveal that the polyphenols, to varying degrees, induce the oligomerization of melittin into larger supramolecular species and concomitantly attenuate the membrane affinity and cytotoxicity of melittin. By investigating the changes in critical properties that regulate the interaction of melittin with cell membranes, we found that the adsorption energy of the polyphenol to melittin and the related change in melittin size are the strongest predictors of the ability of polyphenols to attenuate the membrane affinity and toxicity of melittin. Changes in the hydrophobicity of the protein-polyphenol complexes appear less dominant in driving the reduction in toxicity. The melittin-polyphenol complexes also demonstrate differential resistances to dissociation by a chaotropic agent. These findings demonstrate a small-molecule mediated approach to rationally control the assembly of a model pore-forming peptide on the basis of the biophysical properties of size, hydrophobicity, and adsorption energy.

## Results

### Polyphenols, to Varying Degrees, Attenuate the Affinity and Toxicity of Melittin to Cells

We first sought to quantify the effects of the five polyphenols on the ability of melittin to bind and disrupt cell membranes using SH-SY5Y neuroblastoma cells and the 3-(4,5-dimethylthiazol-2-yl)-2,5-diphenyltetrazolium bromide (MTT) reduction assay, which quantifies mitochondrial function as a readout of cell viability.^31–33^ We leveraged SH-SY5Y cells in this study to focus on neuronal membranes, but it is worth noting that melittin demonstrates similar levels of cytotoxicity to human embryonic kidney cells (HEK293).^15^ Our previous work demonstrated that the treatment of SH-SY5Y cells with an ∼2 μM dose of melittin for 30 min caused an approximately 50% reduction in cell viability, whereas concentrations at or above 5 μM induced complete cell death.^15^

Herein, melittin at 2.5 μM (in monomer equivalents) was incubated in cell culture medium for 1 h at 37 °C under quiescent conditions in the absence and presence of increasing concentrations of five polyphenols from 5 to 50 μM, corresponding to 1:2 to 1:20 molar ratios of melittin-to-polyphenol. Of note, our past research showed that skipping the 1 h incubation step resulted in intermediate changes to the biophysical properties of melittin upon exposure to EGCG concomitant with incomplete neutralization of its toxicity, suggesting strongly that there is a kinetic component to the neutralization mechanism.^14^ Replicating these past experimental conditions where the effects of EGCG against melittin were maximized, we observed that the viability of SH-SY5Y cells exposed to melittin alone for 30 min was reduced to 49 ± 3% (relative to untreated cells, mean ± standard error of the mean (s.e.m.), *P* < 0.001, unpaired *t-*test, Figure 2a). Upon the addition of 50 μM polyphenols (the maximum tested concentration) to melittin for 1 h before cell treatment for 30 min, cell viability was 44 ± 6% relative to untreated cells for apigenin, 53 ± 11% for hispidulin, 73 ± 8% for myricetin, 86 ± 5% for baicalein, and 99 ± 6% for EGCG (Figure 2a–e).

**Figure 2:**
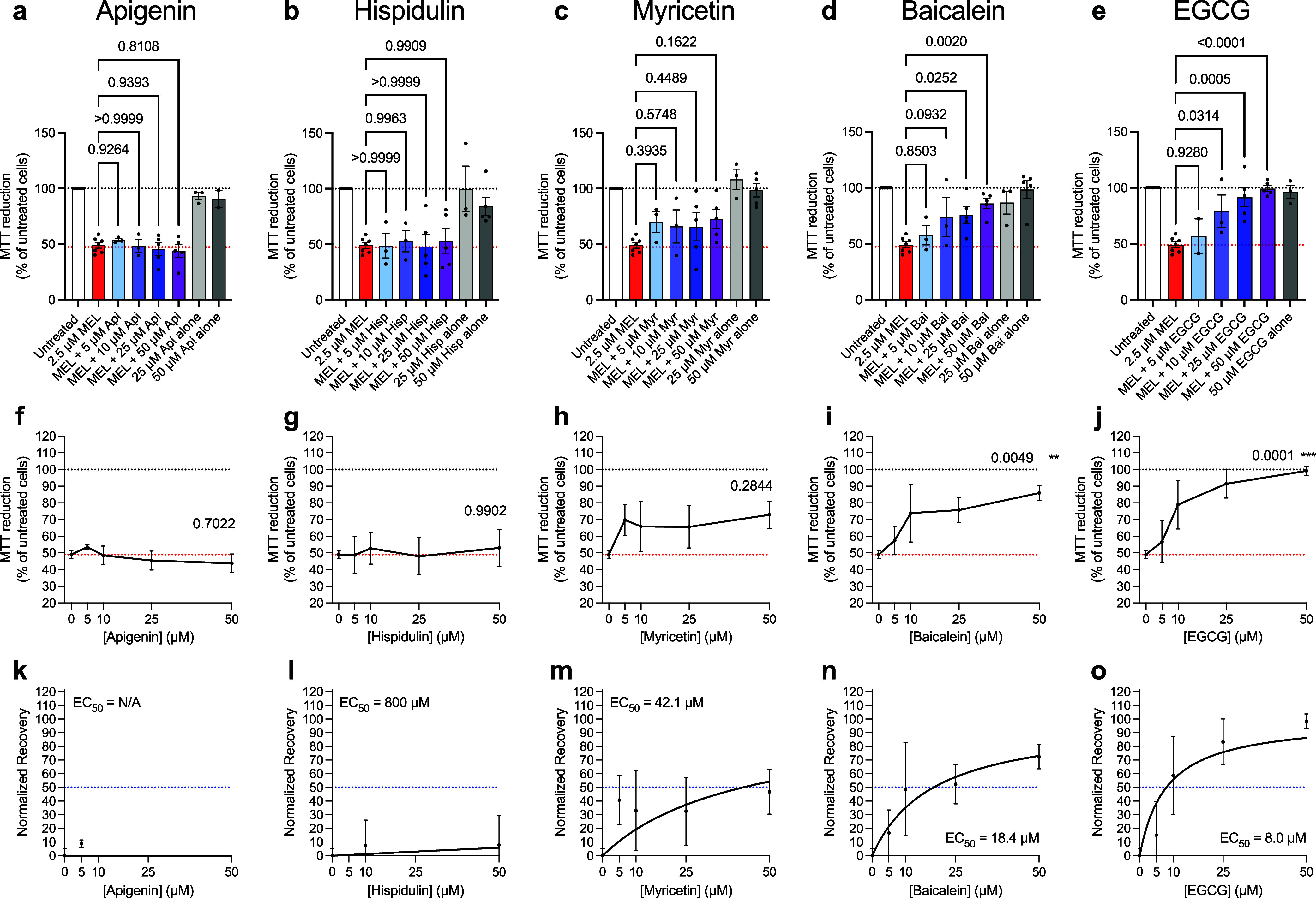
Polyphenols differentially neutralize the cytotoxicity of melittin. MTT reduction assay to assess SH-SY5Y cell viability after treatment with 2.5 μM melittin for 30 min (red bars) and increasing concentrations (shown in darkening colors) of the polyphenols Apigenin (Api, a), Hispidulin (Hisp, b), Myricetin (Myr, c), Baicalein (Bai, d), and epigallocatechin-3-gallate (EGCG, e). Cells treated with the highest concentrations of polyphenols in the absence of melittin are shown (e.g., 25 or 50 μM, gray bars). All samples contained 1% DMSO. In a-e, *P* values were calculated by one-way ANOVA using Dunnett’s post comparison test relative to cells treated with melittin alone. (f–j) Cell viability data analyzed for a treatment effect using one-way ANOVA. Red and gray lines indicate the MTT reduction for cells treated with melittin or cell culture medium (untreated cells) only, respectively. (k–o) Normalized recovery for panels f–j, with melittin treated cells set to 0% and untreated cells to 100%. EC_50_ values corresponding to the half-maximal recovery of cell viability are shown. In all panels, error bars correspond to standard errors of the mean (s.e.m.) of *n* = 2–7 biologically independent experiments. Figures are arranged such that vertical columns are for each drug in the order from left to right of Api, His, Myr, Bai, and EGCG.

A well-defined, dose-dependent increase in cellular recovery was observed for three of the five polyphenol derivatives. Specifically, considering the full series of concentrations tested ranging from 5 to 50 μM, the neutralizing effect was negligible for apigenin (treatment effect: *F*(4,18) = 0.5490, *P* = 0.7022, one-way ANOVA), very low for hispidulin (*F*(4,18) = 0.0701, *P* = 0.9902), modest for myricetin (*F*(4,18) = 1.367, *P* = 0.2844), high for baicalein (*F*(4,18) = 5.401, *P* = 0.0049), and complete neutralization was observed for EGCG (*F*(4,17) = 11.34, *P* = 0.0001) (Figures 2f–j and S2). Data were normalized such that cells treated with melittin alone were 0% and untreated cells were 100%, and EC_50_ values (concentration of polyphenols needed to achieve the half-maximal recovery of cell viability) were calculated as 800 μM for hispidulin, 42.1 μM for myricetin, 18.4 μM for baicalein, and 8.0 μM for EGCG (Figure 2k–o). We omit the EC_50_ value for apigenin herein, as the response was too low to reasonably fit the data. We next sought to assess how the varying efficacies of these polyphenols in neutralizing toxicity may be driven by differences in their chemical structures and interaction affinities with melittin.

### Adsorption Energy Determinations for Melittin and Polyphenols from DFT and AIMD

Density functional theory (DFT) calculations and ab initio molecular dynamics (AIMD) simulations were employed to computationally investigate the interaction between the five polyphenols and melittin. Initially, a single polyphenol molecule was iteratively used as a probe to explore various binding sites on melittin (Figure S3, Table S1). The change of adsorption energy (Δ*E*_ads_) was used as a metric for determining the optimal configuration between each polyphenol and melittin, with greater values of Δ*E*_ads_ relating to a more stable melittin-polyphenol interactome. We observed the highest Δ*E*_ads_ when all aromatic rings of each polyphenol were able to interact with the hydrophilic C-terminal polar amino acid residues of melittin, specifically arginine, lysine, and glutamine (Figure 3). These amino acids displayed the most extensive interactions with the polyphenols at their determined optimal conformations.

**Figure 3:**
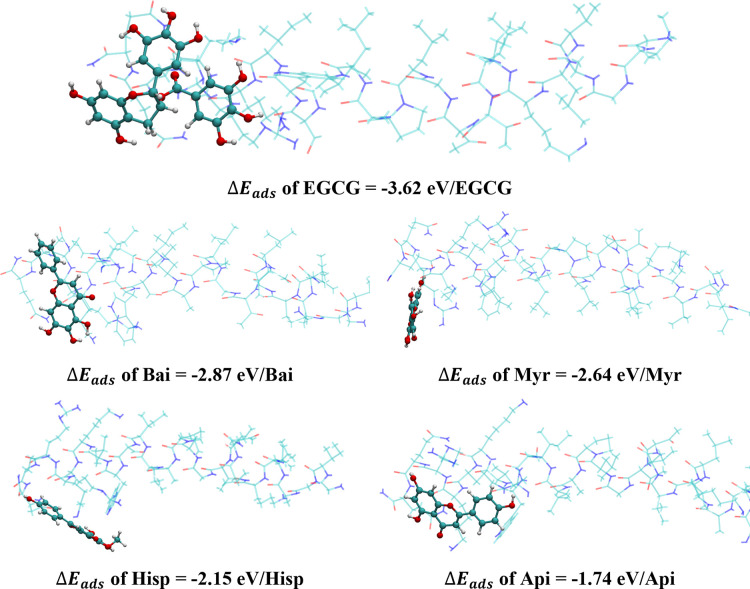
Computational models of melittin-polyphenol complexes. DFT-optimized structures of EGCG, Bai, Myr, Hisp, and Api (with corresponding Δ*E*_ads_) interacting with melittin, computationally annealed from 300 K AIMD simulation (based on the structure/location of conf_6 with highest Δ*E*_ads_ shown in Figure S3). Carbon, oxygen, nitrogen, and hydrogen atoms are represented by green, red, blue, and white balls, respectively. Polyphenol molecules are illustrated using ball-and-stick models for visualization purposes.

The greatest Δ*E*_ads_ was observed with EGCG due to optimized hydrogen bonding and phenyl ring π bond interactions of all three hydroxylated aromatic rings of EGCG with the amino acid side chains of melittin (Figures 3 and S3). These interactions are further facilitated by a conformational rotation at the C2 position of EGCG, allowing its trihydroxyphenyl group to interact favorably with adjacent polar amino acids in the same region. We observed varying degrees of interaction between each ring of the polyphenols and melittin. The trend of increasing Δ*E*_ads_ was closely related to the hydroxylation of each ring. The differential Δ*E*_ads_ was observed with each polyphenol binding to the optimized site on the cationic, C-terminal end of melittin, ranging from −3.62 eV/molecule for EGCG, −2.87 eV/molecule for baicalein, −2.64 eV/molecule for myricetin, −2.15 eV/molecule for hispidulin, and −1.74 eV/molecule for apigenin (Figure 3). Specifically, the hydroxyl groups of the phenyl ring were able to interact with either the carbonyl groups on the glutamine residues or the amino group present at either the end of the polypeptide chain of melittin or its lysine side chains through hydrogen bonding (Figure S4). The trihydroxybenzoyl ring of EGCG exhibited the strongest interactions due to the presence of three hydroxyl groups and their ability to form electrostatic and hydrogen bonding interactions with arginine, lysine, and the two glutamines at the C-terminal end of melittin.

The interaction between a polyphenolic molecule and melittin was most stable, with the greatest Δ*E*_ads_, when the interactions above were optimized, accessible, and utilized. Increasing the number of accessible phenol rings within polyphenols corresponded with unhindered interaction between the phenol rings and the hydrophilic amino acid residues of melittin and is critical for achieving higher Δ*E*_ads_ at the atomistic scale (Figure 3).

### Solvent-Exposed Hydrophobicity Measurements of the Melittin-Polyphenol Complexes

The cytotoxic behavior of proteinaceous pore-forming agents and aggregates of proteins can be enhanced by their small size and increased solvent exposure of hydrophobic regions of amino acids.^14,34,35^ This can be rationalized by the observation that smaller peptides can more readily diffuse to the cell surface, and more hydrophobic peptides and their assemblies can promote their integration into the nonpolar partition of the plasma membrane. In our previous work, we found that the polyphenol EGCG directly influenced the physicochemical properties of melittin, in particular its hydrophobicity and size resulting in the formation of large oligomerized assemblies of melittin.^14^ Considering this previous study and the helical conformation of melittin necessary for it to achieve membrane penetration,^8^ we hypothesized that polyphenols may impact the structure of melittin to varying degrees, which could result in differing capacities to neutralize the toxicity of melittin.

To assess the structural characteristics of melittin in the presence of the polyphenols, we first examined the degree of hydrophobic solvent exposure across the five melittin-polyphenol complexes by means of 8-anilino-1-naphthalenesulfonic acid (ANS) fluorescence binding. Upon binding to solvent-exposed hydrophobic patches on a protein or protein aggregate, a blue shift in the wavelength of maximum fluorescence (λ_max_) of ANS occurs, accompanied by an increase in the fluorescence emission intensity as compared to an unbound dye.^36^ Consistent with past work,^14,15^ we observed that a 10 μM concentration of melittin induced a reproducible blue shift from ∼510 to ∼480 nm and increased the fluorescence intensity of ANS relative to the unbound dye, indicating ANS binding to a solvent-exposed hydrophobic region of melittin (Figure 4a).

**Figure 4:**
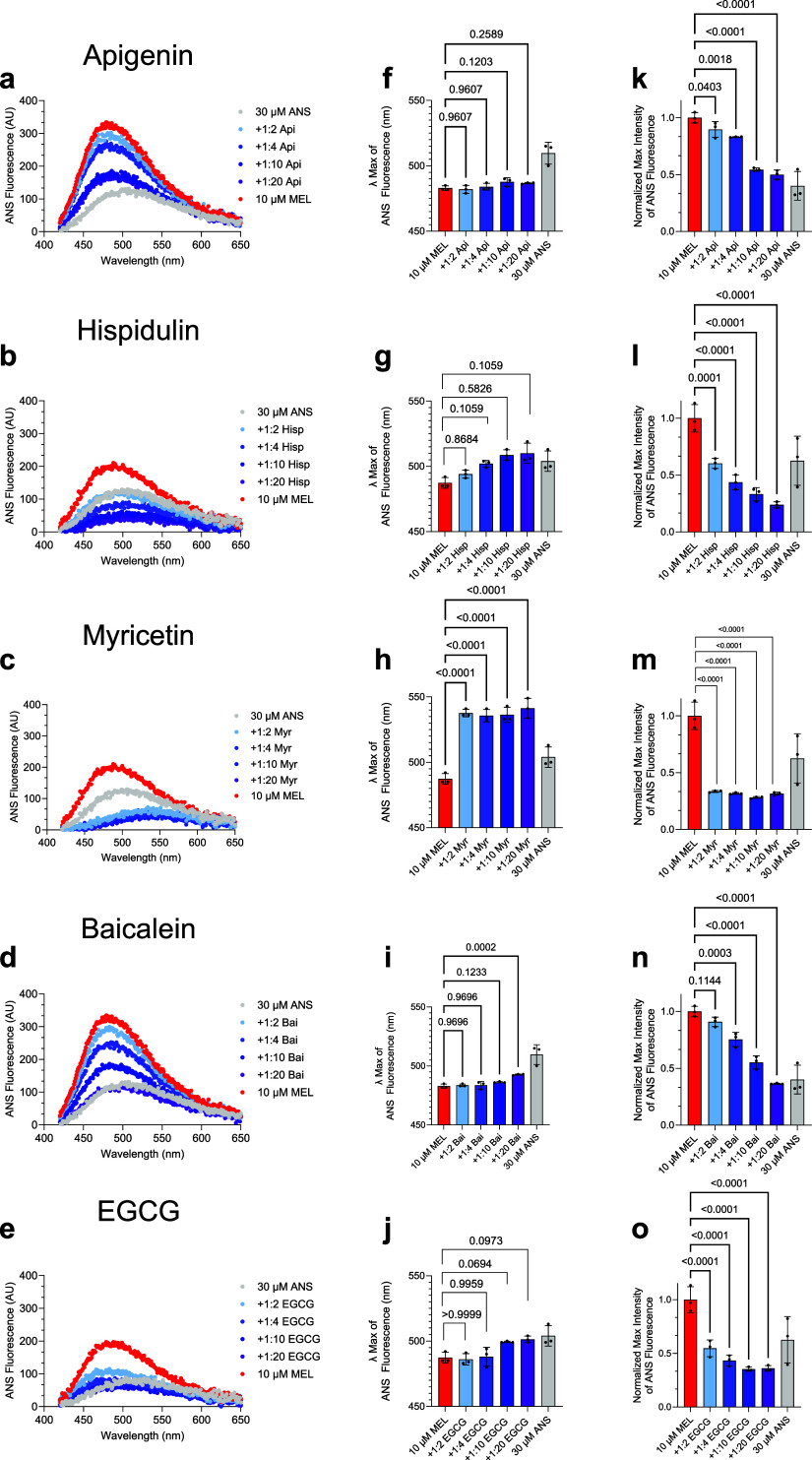
Solvent-exposed hydrophobicity of the melittin-polyphenols complexes. (a–e) 10 μM melittin (red bars) was incubated for 1 h at room temperature with increasing concentrations of select polyphenols (melittin-to-polyphenol ratios of 1:2 to 1:20, shown in different colors), after which time 30 μM 8-anilinonaphthalene-1-sulfonate (ANS) was added to probe the solvent-exposed hydrophobicity of the melittin-polyphenol complexes. Free ANS is shown for reference (gray). (f–j) Wavelength of maximum fluorescence (λ_max_) from (a–e). (k–o) Maximum absorbance intensity from (a–e). Samples containing melittin and select polyphenols were analyzed by one-way ANOVA followed by Dunnett’s multiple comparison test. Error bars indicate the s.e.m. of three technical replicates. Data shown are representative of at least three independent experiments.

Melittin at a concentration of 10 μM was similarly incubated for 1 h at room temperature in the absence or presence of increasing concentrations of the five polyphenols from 20 to 200 μM, corresponding to 1:2 to 1:20 molar ratios of melittin-to-polyphenol, after which time ANS was added from a concentrated stock to a final concentration of 30 μM (Figure 4a–e). We observed a dose-sensitive red shift in the λ_max_ of ANS fluorescence for all the melittin-polyphenol complexes compared to melittin alone, indicating an attenuation in the degree of solvent-exposed hydrophobicity of the melittin species (Figure 4f–j). Similarly, the intensity of maximum ANS fluorescence was dose-dependently attenuated by the polyphenols (Figure 4k–o). We note that myricetin was observed to interfere with the fluorescence of free ANS, limiting its analysis in the presence of melittin (Figures 4h,m and S5). Excluding myricetin, free ANS fluorescence was similar in the presence of the highest concentrations of the other polyphenols (Figure 4a–e and S5). The ANS λ_max_ and max intensity results collectively support that polyphenols decrease the solvent-exposed hydrophobicity of melittin under these conditions. We also assessed hydrophobicity using Nile red fluorescence, finding that these polyphenols all decreased the hydrophobicity of melittin, similar to that observed by ANS fluorescence (Figure S6).

### Polyphenols Increase the Size of Melittin in Solution

We next performed static light scattering (SLS) measurements to quantify the size of melittin in the absence and presence of increasing concentrations of polyphenols (Figure 5a–e), finding that the number of photons backscattered increased as a function of polyphenol identity and concentration. Considering the polyphenols alone (no melittin) in 2% dimethyl sulfoxide (DMSO) demonstrated an increase in light scattering over the baseline signal for buffer alone, especially at concentrations greater than 100 μM, we focused our analyses on the 1:10 molar ratio of melittin-to-polyphenol after subtracting out the signal corresponding to a 10-fold molar excess of each polyphenol alone (Figure 5f). Both analyses show that the scattering intensity of melittin increases as a function of increasing polyphenol concentration, with the size of the melittin-polyphenol complex becoming increasingly larger in the order apigenin, hispidulin, myricetin, baicalein, and EGCG (Figure 5f). Importantly, we confirmed previously that the concentrations of EGCG used herein to induce a large increase in melittin size do not significantly change the secondary structure of melittin, as assessed using circular dichroism spectroscopy.^14^ While this suggests macroscopic changes in secondary structure are not required to induce the oligomerization of melittin by polyphenols, we cannot exclude the potential relevance of more subtle conformational changes that may drive in significant part this mechanism of assembly.

**Figure 5:**
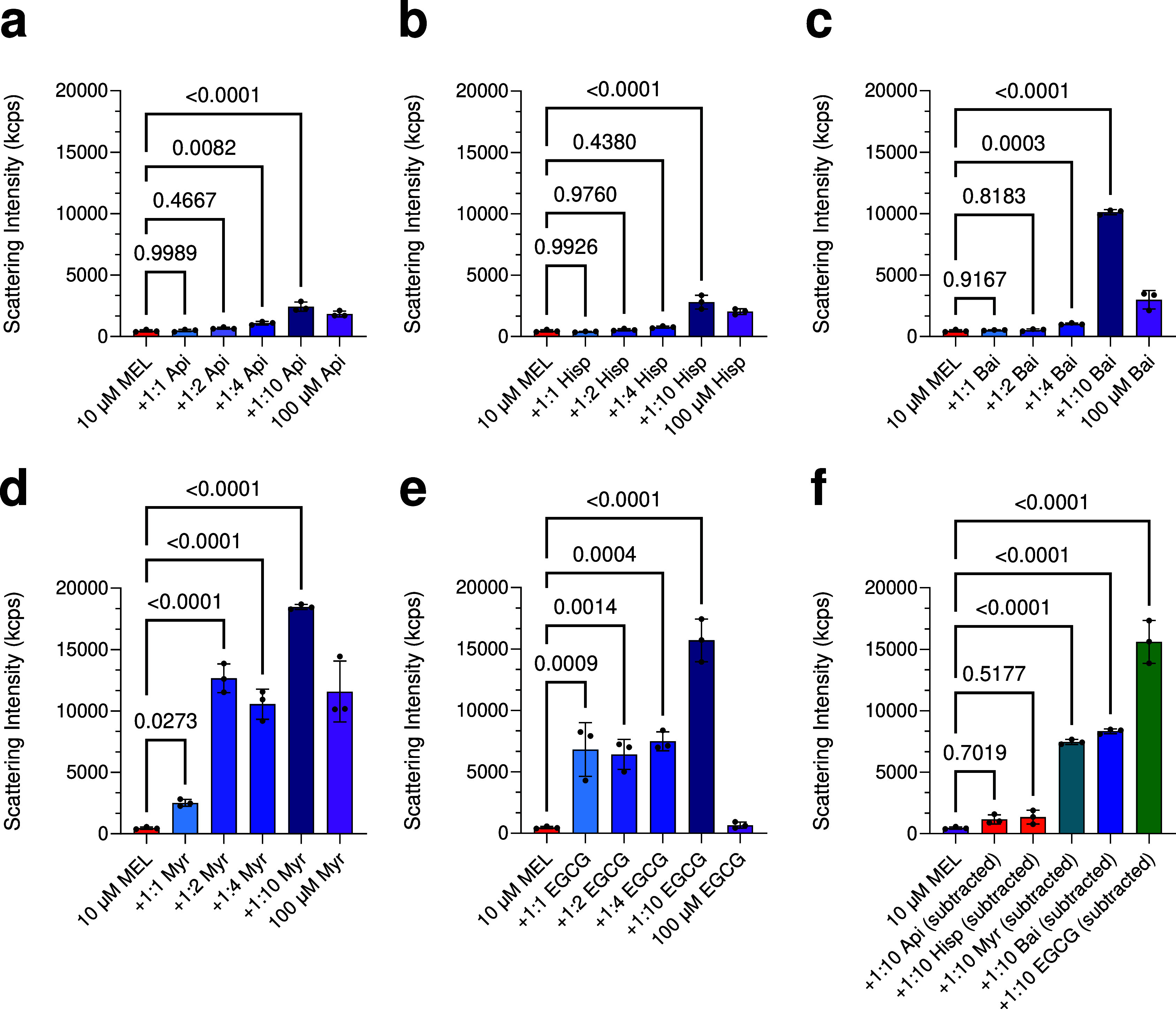
Size of the melittin-polyphenols complexes measured using static light scattering. (a–e) 10 μM melittin was incubated for 1 h at room temperature with increasing concentrations of polyphenols (MEL-to-polyphenol ratios of 1:0 to 1:10). 100 μM of each polyphenol was tested for reference (purple). (f) Conditions containing a 1:10 molar ratio of melittin-to-polyphenol were subtracted against the background signal for 100 μM polyphenol. Samples containing melittin and polyphenols were analyzed by one-way ANOVA followed by Dunnett’s multiple comparison test. Error bars indicate the s.e.m. of three technical replicates. Data were confirmed in at least two independent experiments.

We leveraged the size of the melittin-polyphenol complexes to validate the existence of the larger species. Solutions of melittin (10 μM) or melittin incubated as above with a 10-fold molar excess of EGCG were passed through a 0.22 μm filter, and unfiltered solutions and their related flowthroughs were analyzed using turbidity absorbance. Filtration lowered the absorbance signal of the melittin-polyphenol complex (Figure S7a), as larger species were unable to pass through a 0.22 μm filter. Free EGCG at a commensurate concentration was also tested and observed to have a signal which reduced upon filtration (Figure S7b), albeit to a lesser extent in comparison to the melittin-polyphenol complex, which limited the use of turbidity to assess the size of the complexes in solution and shows some free EGCG was likely retained on the filter in the previous experiment. Lastly, we observed this filtration did not visibly change the absorbance signal for 10 μM melittin (Figure S7c) or 10 μM melittin with 2% DMSO (as in the assays containing polyphenols, Figure S7d), suggesting the solutions do not contain large assemblies in the absence of polyphenols.

### Cytotoxic Attenuation Best Correlates with an Increase in Melittin Size

Previous studies have shown that solvent-exposed hydrophobicity and size can play important roles in the cytotoxicity of pore-forming toxins and protein aggregates.^14,35,37^ Our results provide further evidence that melittin experiences an increase in size that occurs through its interaction with polyphenols, sequestering melittin into less- or nontoxic assemblies, depending on the polyphenol and concentration employed. We therefore sought to rationalize the relationship between polyphenol treatment and changes in melittin structure with their ability to induce cellular dysfunction through their interaction with the cell surface.

Considering past studies analyzing the relationship between size and hydrophobicity with toxicity revealed linear correlations between these biophysical parameters,^35^ we attempted similar analyses herein. In doing so, we found that for the conditions where polyphenols effectively neutralized the toxicity of melittin (e.g., 1:10 and 1:20 molar ratios of melittin-to-polyphenol), a clear correlation existed across the polyphenols between their neutralization ability and changes in the energy of adsorption (Δ*E*_ads_) of the polyphenols to melittin, as determined from the computational studies (Figure 6a). Complexes that show larger negative changes in adsorption energy exhibit a stronger correlation with normalized recovery (*R* ^2^ = 0.6598, *P* = 0.0495 for a linear fit, Figure 6a). Therefore, the direct interaction of polyphenols to melittin is correlated with their neutralizing ability. The hydrophobicity of melittin had a modest, nonsignificant, and positive correlation with cell viability when analyzed by λ_max_ (*R* ^2^ = 0.0623, *P* = 0.6224 for a linear fit, Figure 6b, see Figure S8a for further polyphenol doses) or a modest, nonsignificant negative correlation with normalized maximum intensity of ANS fluorescence (*R* ^2^ = 0.1892, *P* = 0.3886 for a linear fit, Figure 6c, see Figure S8b for further polyphenol doses). The attenuation of melittin cytotoxicity by polyphenols proved to display a very strong correlation with the size of the melittin-polyphenol complexes (*R* ^2^ = 0.9697, *P* = 0.0003 for a linear fit, Figure 6d, see Figure S8c,d for further polyphenol doses). Therefore, the biophysical parameters studied herein correlate with toxicity to varying degrees, where the neutralization of melittin toxicity is connected to Δ*E*_ads_ (*P* < 0.05) and strongly associated with its size (*P* < 0.001).

**Figure 6:**
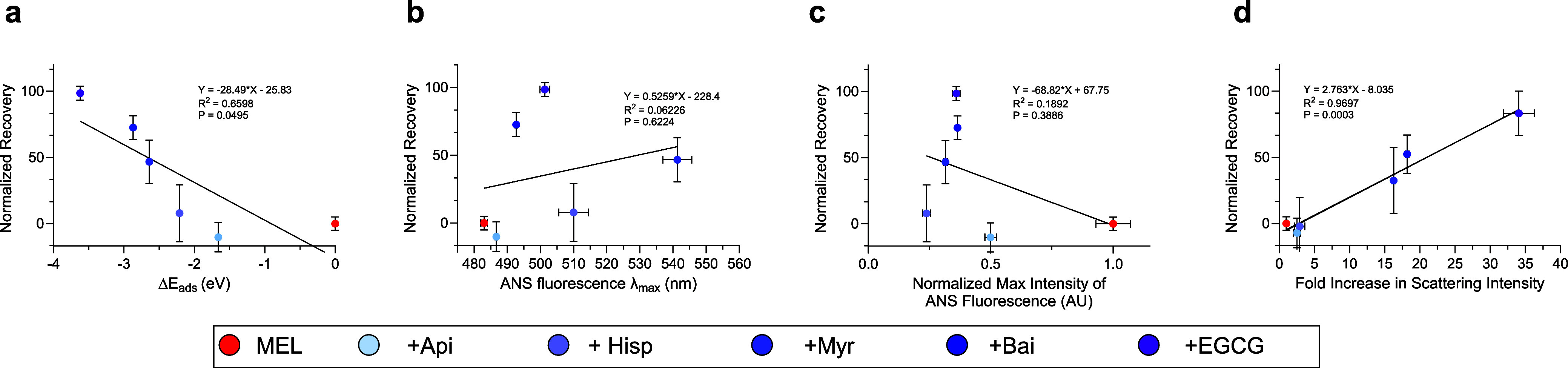
Relationships between normalized recovery of cell health and the biophysical characteristics of melittin-polyphenol complexes. Correlation of the measured biophysical properties for melittin in the absence and presence of the polyphenols. (a) Normalized recovery versus Δ*E*_ads_ for 1:20 molar ratios of melittin-to-polyphenol. (b) Normalized recovery versus ANS λ_max_ for 1:20 molar ratios of melittin-to-polyphenol. (c) Normalized recovery versus normalized maximum ANS fluorescence intensity for 1:20 molar ratios of melittin-to-polyphenol. (d) Normalized recovery versus the fold increase in static light scattering for 1:10 molar ratios of melittin-to-polyphenol. In all panels, error bars indicate s.e.m. of three technical replicates.

With the apparent importance of the size of the melittin-polyphenol complexes and that static light scattering was a facile way to screen molecules for an increase in size, we next examined four additional molecules alongside EGCG as a positive control. Specifically, we tested flavone (scaffold with no hydroxylation), 7-hydroxyflavone (monohydroxylation), catechin (disubstituted on two phenol rings), and morin (structurally similar to catechin). As was predicted, flavone and 7-hydroxyflavone caused small increases in the size of the melittin-polyphenol complexes, and catechin and morin caused larger and similar changes in light scattering. As in the other tests, EGCG was able to most potently induce the oligomerization of melittin, leading to the largest formed complexes with melittin (Figure S9). These results support static light scattering as a pragmatic way to efficiently screen polyphenols in future studies.

### Assessment of the Stabilities of the Melittin-Polyphenol Complexes

Leveraging static light scattering, we next sought to determine the relative stabilities of the various melittin-polyphenol complexes. Focusing on the four polyphenols that were fully characterized above and showed efficacy in reducing the toxicity of melittin to cells (hispidulin, myricetin, baicalein, and EGCG), we monitored melittin-polyphenol complex size while titrating in increasing amounts of the chaotropic agent guanidine hydrochloride, which dissociates protein complexes by disrupting hydrogen bonds, van der Waals forces, and ionic interactions. All of the melittin-polyphenol complexes were progressively dissociated with increasing concentration of guanidine hydrochloride, with differing measured EC_50_ values for dissociation of 0.22 M for hispidulin, 0.31 M for myricetin, 0.44 M for EGCG, and 0.54 M for baicalein (normalized data in Figure 7, raw data in Figure S10). These results support that the melittin-polyphenol complexes have differing resistances to dissociation in solution.

**Figure 7:**
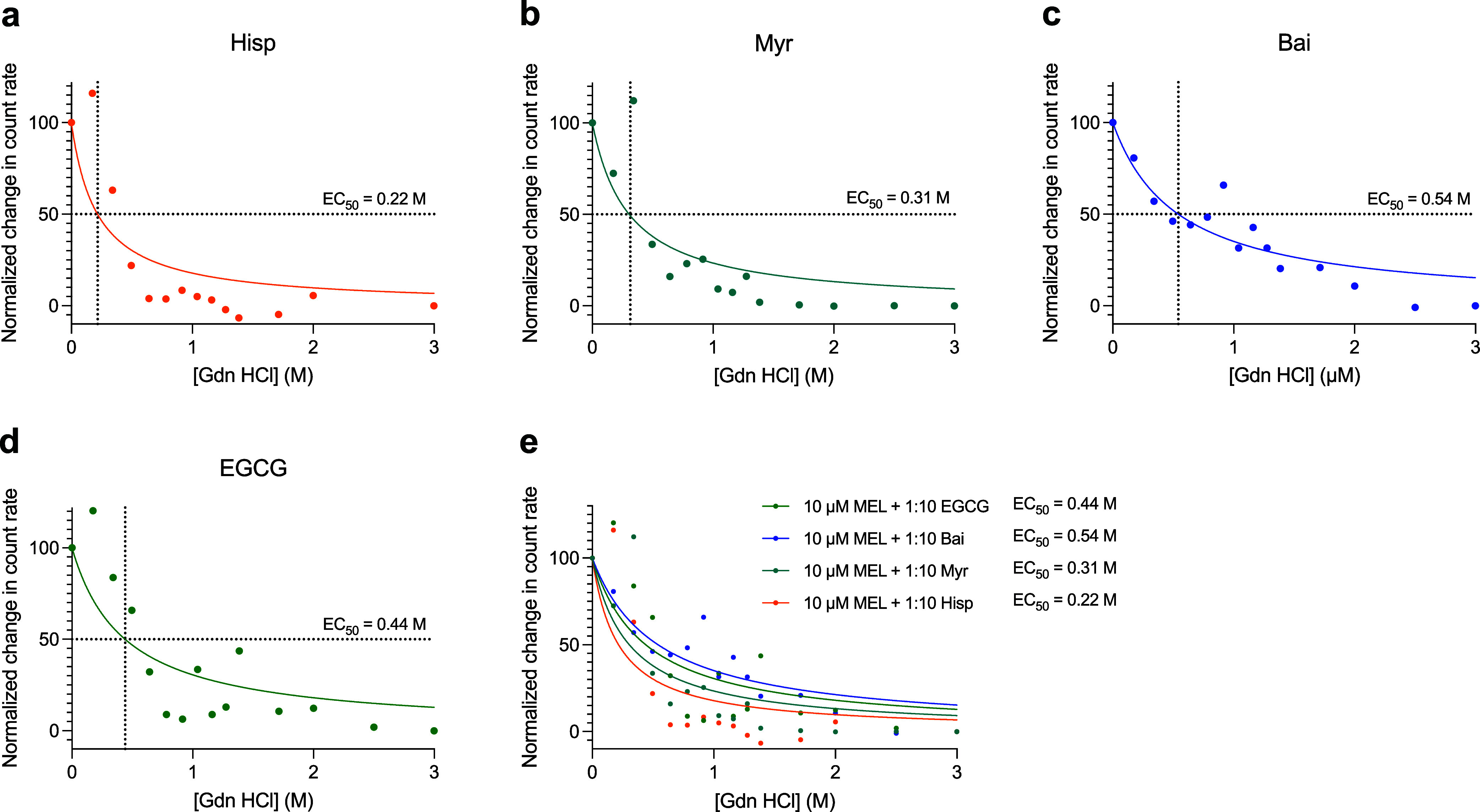
Resistance to dissociation for the melittin-polyphenol complexes. The polyphenols that attenuated the cytotoxicity of melittin were incubated for 1 h in the presence of a 10-fold molar excesses of Hisp (a), Myr (b), Bai (c), or EGCG (d) and measured using static light scattering. (e) Comparison of the dissociation curves for the above melittin-polyphenol complexes. The resulting count rate was monitored as the chaotropic agent guanidine hydrochloride (Gdn HCl) was titrated in from 0 to 3 M Gdn HCl. Raw data are shown in Figure S10. Data were normalized such that 0 M Gdn HCl was set to 100% and 3 M Gdn HCl was set to 0%. Resulting EC_50_ values were determined using fits for [Inhibitor] vs normalized response (GraphPad Prism).

## Discussion

Polyphenols are a well-researched class of plant-derived natural compounds, recognized for their ability to influence pathways related to cardiovascular diseases, neurodegenerative disorders, cancer, and diabetes.^38^ For aggregation prone peptides related to neurodegenerative diseases, they have been utilized to control peptide assembly, including stabilizing nontoxic misfolded protein oligomers, which has facilitated research on Alzheimer’s disease.^39^ Previous studies highlighted the significance of the polyphenol EGCG, known for its antioxidant, anti-inflammatory, and antiviral properties, as a potential countermeasure against melittin.^14^ Building on this work, we examined the effects of four additional structurally similar polyphenols (apigenin, hispidulin, myricetin, and baicalein) to better understand the relationship between the biophysical properties of a toxic peptide and its ability to induce cellular dysfunction, particularly in the context of small molecule-induced protein assembly and the ability of this mechanism to regulate the membrane affinity and toxicity of pore-forming toxins.

The ability of melittin to bind cell membranes and form pores is associated with its amphipathic and hydrophobic nature. In its membrane-active secondary structure, melittin adopts an α-helix,^8^ and its proline-hinge motif disrupts hydrogen bonding and induces a bend in the melittin structure.^40,41^ Upon interacting with cell membranes, the cationic, hydrophilic C-terminal region of melittin can remain aligned with negatively charged phospholipid head groups as its hydrophobic N-terminal region penetrates the lipid bilayer core, enabling its pore-forming capabilities.^7^ This disrupts the natural organization of the bilayer and can lead to pore formation.^8,11,12,14,15^

The five polyphenols examined in this study demonstrate varying capacities to alter the biophysical properties and cytotoxic effects of melittin. For the melittin-polyphenol complexes, toxicity was observed to correlate weakly with hydrophobicity, well with Δ*E*_ads_, and strongly with supramolecular size. These results suggest that a more potent ability of polyphenols to form stable interactions with melittin leads to a larger overall size of the melittin-polyphenol complexes, enabling a greater reduction in the toxicity of melittin through its predominant sequestration into membrane-inactive species. The ratio of melittin to lipid is important in its ability to form transmembrane pores, and not simply the absolute concentration of melittin.^8^ Our results add support to this postulation, as the dose- and polyphenol-dependent induction of melittin oligomerization proportionally reduces the ratio of melittin interacting with the lipidic component of the membrane, therein also dose-dependently impacting its cytotoxicity. The stabilized melittin-polyphenol complexes also demonstrate differential resistances to dissociation, as assessed using the strong chaotropic agent guanidine hydrochloride. This supports that the melittin-polyphenol complexes are driven by a combination of noncovalent interactions, including hydrogen bonds, van der Waals forces, and ionic interactions. The results of our computational work highlight the importance of hydrogen bonding in the interaction of polyphenols with melittin, in particular. While it is clear that polyphenols induce the supramolecular oligomerization of melittin, leading to differential biological activities, future work is necessary to better understand the mechanism underpinning the formation of the melittin-polyphenol complex, as well as the specific stoichiometries of binding for each polyphenol to melittin.

The presence of a chromone backbone with an attached C2 phenyl group is a notable characteristic shared among the planar polyphenols apigenin, hispidulin, myricetin, and baicalein. The diverse array of chromone structures is of considerable interest to researchers due to the tunability of their biological activity based on substitution patterns.^42^ The variations in hydroxylation of the C2 phenyl group, along with modifications to the backbone of each polyphenol itself, to some degree, appear to impact its ability to interact with melittin and mitigate its cytotoxic effects. Among the polyphenols, EGCG stands out as it is not fully planar and features an additional trihydroxybenzoyl substituent group at C3 of its chromanone backbone. These two distinct attributes facilitate dynamic conformational adjustments that likely optimize the ability of EGCG to participate in hydrogen bonding and π–π interactions with melittin. The nonplanar, multiring EGCG structure increases the number and strength of π-bond interactions with the positively charged amino acid side chains of melittin. Its rotational flexibility facilitates optimal positioning for hydrogen bonding and electrostatic interactions (Figure S3), contributing to a greater Δ*E*_ads_ compared to other polyphenols. Unlike planar polyphenols, for instance, the aromatic rings engage in stacking interactions with arginine and lysine residues, while the hydroxyl groups provide electrostatic complementarity to the polar regions of melittin. In contrast, the other four polyphenols, although they vary in degrees of hydroxylation and types of substitutions, all have a planar chromone backbone that lacks rotational flexibility, which limits their binding efficiency. These findings emphasize the structural advantages of EGCG and highlight the supportive role of flexible aromatic rings in stabilizing melittin-polyphenol complexes.

In the context of cancer, the potential of melittin-polyphenol complexes to combat metastatic tumors requires further exploration. Melittin itself is widely recognized to cause increases in caspase-3 expression resulting in apoptosis, disruption of cellular matrix formation, cell cycle arrest, and attenuated cellular migration, all contributing to a reduction in cancer proliferation.^43–45^ Furthermore, melittin-associated pore formation releases damage-associated molecular patterns (DAMPs) into the extracellular space leading to downstream production of key pro-inflammatory cytokines, such as tumor necrosis factor (TNF) and interleukin-2 (IL-2), by T cells.^46^ However, the inherent hemolytic activity and unstable nature of melittin limits its current use, necessitating exploration of modified melittin complexes.^43–45^ Stabilization of melittin via polyphenols offers a potential solution where pore formation could be restricted, time-dependent, reversible, and tuned to specific desired effects in targeted cancerous tissue. In conjunction with this, the introduction of an adjuvant or other immunomodulatory small molecule may elicit improved immune signaling in tumor-induced immune-privileged tissues. This has been previously demonstrated in vivo with a nanocarrier-based tannic acid-Fe^3+^-melittin-adjuvant conjugate, where primary tumors shrank and distal growth was affected by immune upregulation.^45^ Ongoing efforts, as this work describes, aim to optimize the stability of melittin to improve bioavailability and enable tissue-specific targeting that may have multifaceted roles in immune stimulation, including with chronic disease.^47^

EGCG and melittin assemblies and metal polyphenol networks (MPNs), the supramolecular networks formed from polyphenols and multivalent metal ions, have been extensively explored for a wide range of anticancer, gene therapy, bioimaging, and surface functionalization applications.^45,48,49^ These assemblies add a valuable tool for nanomaterials and nanomedicine. Polyphenols hold promise as antimicrobial coatings, for example in bandages. In addition to melittin, EGCG can also attenuate the toxicity of α-hemolysin, a markedly larger pore-forming protein from *Staphylococcus aureus* with a molecular weight of 33.2 kDa in its monomeric form and 232.4 kDa in its homoheptameric, β-barrel form that can penetrate cell membranes.^14,50^ Given similarities between melittin and other pore-forming toxins from venomous species such as ants, cnidarians, spiders, bees, and wasps, polyphenols may also contribute to antivenom treatments in the acute care setting. The quantification of the affinity of the various polyphenol molecules with melittin and correlation with the relative size of supramolecular protein assembly and consequent cell viability in this study has interesting implications for material design. With estimates of polyphenol binding affinity with various pore-forming toxins, surface functionalization with a rationally designed mixture of polyphenols, with energetically tailored targets, might address a much broader range of possible pathogens and toxins for wound dressings and medical device coatings.^51^ This approach might be useful as an antibiofilm strategy to mitigate infection in complex chemical and biological environments. Understanding the relative binding energies of different polyphenols with pore-forming proteins, as well as metal ions for MPNs, might also be applied to designing tailored mixtures of vesicles that respond to specific targets and release the corresponding polyphenol for multimodal approaches to disease and pathogen threats in vivo.^48^ Broadly, these findings suggest a strategy to engineer membrane-interfacing materials that attenuate the membrane–disruptive activity of melittin, suggesting the potential of polyphenols for protective therapeutic coatings for biomaterial surfaces or in the creation of delivery systems with tuned release kinetics, among other nanomaterial and nanomedicine applications.

EGCG provided an early example of an Aβ,^52^ α-synuclein,^52^ and tau^53^ protein aggregation inhibitor by remodeling amyloid fibrillar forms of these proteins and stabilizing unique aggregates from their monomeric forms, resulting in the formation of off-pathway, stabilized intermediate species. The poor pharmacokinetic properties of EGCG limited its use in humans,^53,54^ emphasizing the likely need for a diverse set of polyphenols to control pore-forming toxin assembly in complex biological milieu in vivo. The translatability of this strategy depends on finding polyphenols that are pharmacokinetically and metabolically pragmatic. In this context, polyphenols have been studied extensively in a variety of clinical trials, in many cases in specific foods or food extracts, and have shown a wide variety of health benefits.^55–57^ Nonetheless, further research is necessary to assess if the melittin-polyphenol oligomerization strategy proceeds in a similar fashion in vivo. Furthermore, our results support that the melittin-polyphenol complexes can have differing stabilities, as assessed by their resistance to dissociation. The combination of differential size changes that could be induced by diverse arrays of polyphenols beyond those in this manuscript, alongside the differing stabilities of these induced complexes, lends this system well to optimization in future work.

## Conclusion

We show that polyphenols can drive the assembly of the pore-forming toxin melittin into polyphenol-dependent supramolecular complexes of varying degrees of cytotoxicity, binding energies, hydrophobicities, sizes, and resistances to denaturation. Our analyses provide fundamental insight into the relevance of these physicochemical properties that impact pore-forming agent toxicity and bioactivity. They, moreover, support the existence of a size-hydrophobicity-cytotoxicity relationship for melittin, showing in particular the ability of polyphenols to form larger, stable supramolecular complexes with melittin is a driving factor in reducing their cytotoxicity. This mechanism provides a promising avenue for mitigating the membrane affinity and associated cytotoxic effects of melittin and related pore-forming peptides by controlling their assembly using cost-effective, low-toxicity polyphenols and affords a framework to rationally design small molecule-stabilized biomaterials that can regulate interfacial interactions, with relevance to nanomaterials and nanomedicine.

## Experimental Methods

### Reagents

Apigenin, hispidulin, myricetin, and baicalein (>95%) were acquired from Sigma-Aldrich (MO, USA). Aliquots were prepared at a concentration of 5 or 10 mM in DMSO and stored at −20 °C. EGCG (>95%) was acquired from Sigma-Aldrich (MO, USA). Aliquots were prepared at a concentration of 1, 5, or 10 mM in water or DMSO and stored at −20 °C. Melittin (>85%) was acquired from Sigma-Aldrich (MO, USA) and aliquots were prepared at a concentration of 1 mM in water and stored at −20 °C. Samples containing pore-forming agents were handled and disposed of with care and according to the manufacturer’s recommendations and guidelines. All samples containing proteins were prepared or stored in Eppendorf LoBind Tubes (Hamburg, Germany).

### Neuroblastoma Cell Culture

Human SH-SY5Y neuroblastoma cells (ATCC, VA) were cultured in Dulbecco’s modified Eagle’s medium (DMEM)/F-12 with l-glutamine, *N*-(2-hydroxyethyl)­piperazine-*N*-ethanesulfonic acid (HEPES), and phenol red (11330032, ThermoFisher Gibco, MA) and supplemented with 10% fetal bovine serum (FBS) and 1.0% antibiotics (penicillin–streptomycin, ThermoFisher Gibco, MA). Cell cultures were maintained in a 5% CO_2_-humidified atmosphere at 37 °C and grown until they reached 80% confluence for a maximum of 20 passages.^14,15^

### MTT Reduction Assays

All samples for cell experiments were maintained at 1% DMSO. Melittin (2.5 μM, in monomer equivalents) was added to cell culture medium in the absence of cells and incubated with or without increasing concentrations of apigenin, hispidulin, myricetin, baicalein, and EGCG for 1 h at 37 °C under quiescent conditions, and then added to SH-SY5Y cells seeded in 96-well plates for 30 min. Upon aspirating the cell culture, the conditions were incubated in 0.5 mg/mL 3-(4,5-dimethylthiazol-2-yl)-2,5-diphenyltetrazolium bromide (MTT, purchased from Sigma-Aldrich, MO, USA, dissolved in Dulbecco’s phosphate-buffered saline (DPBS) and prewarmed to 37 °C) for 4 h at 37 °C under quiescent conditions.^14,15^ Upon lysing cells with stop solution (100% DMSO), the absorbance of formazan crystals was recorded using a CLARIOstar Plus plate reader (BMG Labtech, Ortenberg, Germany) at 590 nm. Cell viability was expressed as the percentage of MTT reduction in treated cells as compared to untreated cells.

### ANS Binding Measurements

Samples for all the biophysical experiments were maintained at 2% DMSO. Solutions with melittin (10 μM, in monomer equivalents) in DPBS were aliquoted after incubation for 1 h at room temperature in the absence or presence of apigenin, hispidulin, myricetin, baicalein, and EGCG up to 200 μM, and 30 μM 8-anilinonaphthalene-1-sulfonate (ANS, Sigma-Aldrich, MO, USA) was subsequently added from a 1.5 mM concentrated stock.^14,15^ Emission spectra were recorded using a plate reader (ClarioStar Plus, BMG Labtech, Ortenberg, Germany) with excitation at 380 nm and emission measured from 420 to 650 nm. Spectra were background subtracted to the buffer alone.

### Nile Red Binding Measurements

Experiments were performed as described for ANS fluorescence. Nile red (acquired from Sigma-Aldrich) was prepared in 100% DMSO at 5 mM and subsequently added to solutions (at a final concentration of 5 μM Nile red) containing 10 μM melittin in the absence or presence of various concentrations of polyphenols. Emission spectra were recorded using a plate reader (ClarioStar Plus, BMG Labtech, Ortenberg, Germany) with excitation at 530 nm and emission measured from 560 to 700 nm.

### Turbidity Absorbance

Samples were analyzed for absorbance using a plate reader (ClarioStar Plus, BMG Labtech, Ortenberg, Germany) with spectral scanning from 350 to 650 nm.

### Static Light Scattering

Solutions with melittin (10 μM, in monomer equivalents) in dPBS were incubated for 1 h at room temperature in the absence or presence of apigenin, hispidulin, myricetin, baicalein, and EGCG up to 200 μM. Static light scattering measurements were performed with fixed parameters for attenuator (attenuator 10 for dissociation experiments or 11 for sizing measurements), as determined from the sample of melittin, and cell position of 4.65 mm at 25 °C using the Malvern Zetasizer Nano S instrument (Malvern Panalytical Ltd., Malvern, UK). A low volume (70 μL) disposable cuvette was used (BRAND, Wertheim, Germany).

### Resistance to Dissociation with Guanidine Hydrochloride

Guanidine hydrochloride (Sigma-Aldrich) was prepared in 20 mM phosphate buffer at a concentration of 6 M. Solutions were prepared as described above with 10 μM melittin and a 10-fold molar excess of Myr, His, Bai, and EGCG. Gdn HCl was slowly titrated in while monitoring the count rate via static light scattering, with Gdn HCl concentrations spanning from 0 to 3 M.

### Statistics

Comparisons between the different groups were performed by one-way ANOVA followed by Dunnett’s post comparison test and the unpaired, two-tailed Student’s *t*-test, and all statistical tests were performed in GraphPad Prism 10.4 (CA, USA). *P* < 0.05 was accepted as statistically significant.

## Computational Details

### Density Functional Theory (DFT) Calculations

DFT calculations were performed using the Perdew–Burke–Ernzerhof (PBE) version of the generalized gradient approximation (GGA), as implemented in the CP2K software package.^58,59^ A 400 Ry cutoff was used to describe the electrostatic terms. The Goedecker–Teter–Hutter (GTH) pseudopotentials were selected for treating core electrons, while the valence electron states were calculated with double-ζ Gaussian basis sets.^60,61^ Long-range dispersion (van der Waals) corrections were applied using Grimme’s DFT-D2 method.^62^ The lattice parameters of simulation cell containing melittin structure (adopted from PDB ID: 2MLT) were set as *a* = 50.00 Å, *b* = 50.00 Å, *c* = 30.00 Å (α = β = γ = 90°), providing a minimum distance of 12 Å between periodic images. Sampling the Brillouin zone with the Γ-point was adequate, considering a relatively large size of simulation cell. Geometry optimization of bare melittin (consisting of 428 atoms) was initially carried out using the Broyden–Fletcher–Goldfarb–Shanno (BFGS) algorithm, allowing relaxation of the atomic coordinates for all atoms.

### Computational Adsorption Energy

The DFT adsorption energy of adsorbed structures (Δ*E*_ads_; eV/molecule) on melittin at 0 K was calculated as: Δ*E*_ads_ = *E*_adsorbed_ – *E*_melittin_ – *E*_molecule_. *E*_adsorbed_ defines the energy of a molecule adsorbed on melittin, while *E*_melittin_ is the energy of bare melittin. *E*_molecule_ is the energy of a single molecule in the gas phase.

### Molecular Dynamics Simulations

DFT-based ab initio molecular dynamics (AIMD) simulations were also conducted in the canonical constant-temperature, constant-volume ensemble (*NVT*) using a 1.0 fs time step and a Nosé-Hoover chain thermostat at 100 and 300 K. Well-equilibrated MD trajectories were sampled for at least 10 ps at 100 K and continuously for at least 20 ps at 300 K for the following adsorbed systems: (1) EGCG, (2) baicalein, (3) myricetin, (4) hispidulin, and (5) apigenin adsorbed on melittin. The structure features of conf_6 (shown in Figure S3) were adopted for all polyphenol species and subjected to AIMD simulations due to their highest Δ*E*_ads_. The selected configurations of AIMD-perturbed adsorbed structures were annealed further to 5 K for each molecule, subsequently followed by geometry optimization to obtain the representative structures at 0 K (shown in Figure 3).

## Supplementary Material



## Data Availability

Data supporting the findings of this manuscript are available from the corresponding authors upon request.

## Notes

Figure S1. Chemical structures of the studied polyphenols. Figure S2. Additional comparison of the neutralizing effects of the five tested polyphenols against melittin toxicity. Figure S3. DFT-optimized structures of a single EGCG molecule adsorbed on the different sites of melittin, along with the corresponding Δ*E*_ads_ (without AIMD simulations). Figure S4. Interaction EGCG and melittin highlighting adsorption energy trends. Figure S5. ANS Fluorescence curves of polyphenols alone. Figure S6. Nile red fluorescence measurements of melittin-polyphenol complexes. Figure S7. Size comparisons before and after filtration. Figure S8. Expanded relationships between normalized recovery of cell health and the biophysical characteristics of melittin-polyphenol complexes from Figure 6. Figure S9. Additional polyphenols screened for size using static light scattering. Figure S10. Raw data for Gdn HCl dissociation experiments on the stabilized melittin-polyphenol complexes. Table S1. Cartesian coordinates (in Å) of DFT-optimized structure of melittin interacting with single EGCG molecule (PDF)
