# Supplementary material for: Polyphenol–Mediated Peptide Assembly Modulates Melittin Toxicity: A Structure–Activity Strategy for Neutralizing the Interface Affinity of Pore–Forming Toxins to Cell Membranes
Source: ACS Appl Mater Interfaces. 2025 Aug 25;17(36):51407–20. doi: 10.1021/acsami.5c09472 (PMC12442020; doi:10.1021/acsami.5c09472)
Supplement: Supplementary file 1 [file am5c09472_si_001.pdf]

Supporting Information

Polyphenol–mediated peptide assembly modulates melittin toxicity: A structure–activity strategy for neutralizing the interface affinity of pore–forming toxins to cell membranes

Hojin Han[1,+], Julia C. Palchak[1,+], Johnathan R. Pinc[1,+], Michael Nguyen[1], Keira A. Atchley[1], Donald J. Darrell[1], Elaine S. Kim[1], Patrick H. Lee[1], Mary Claire Schleck[1], Alyssa R. Cornell[1], Joseph E. Buselmeier[1], Kate M. Bacon[1], Kaitlyn Zang[1], Nathan Burpeau[1], Justus M. Gabriel[1], Carol A. Anderson[1], F. John Burpo[1,2], Lucas B. Fallot[1,2,*], Simuck F. Yuk[1,2,*], and Ryan Limbocker[1,2,*]

[1]Department of Chemical and Biological Science and Engineering, United States Military Academy, West Point, NY 10996, United States of America.

[2]Photonics Research Center, United States Military Academy, West Point, NY 10996, United States of America.

+These authors contributed equally.

*Correspondence to: lucas.fallot@westpoint.edu (L.B.F.), simuck.yuk@westpoint.edu (S.F.Y.) or ryan.limbocker@westpoint.edu (R.L.)

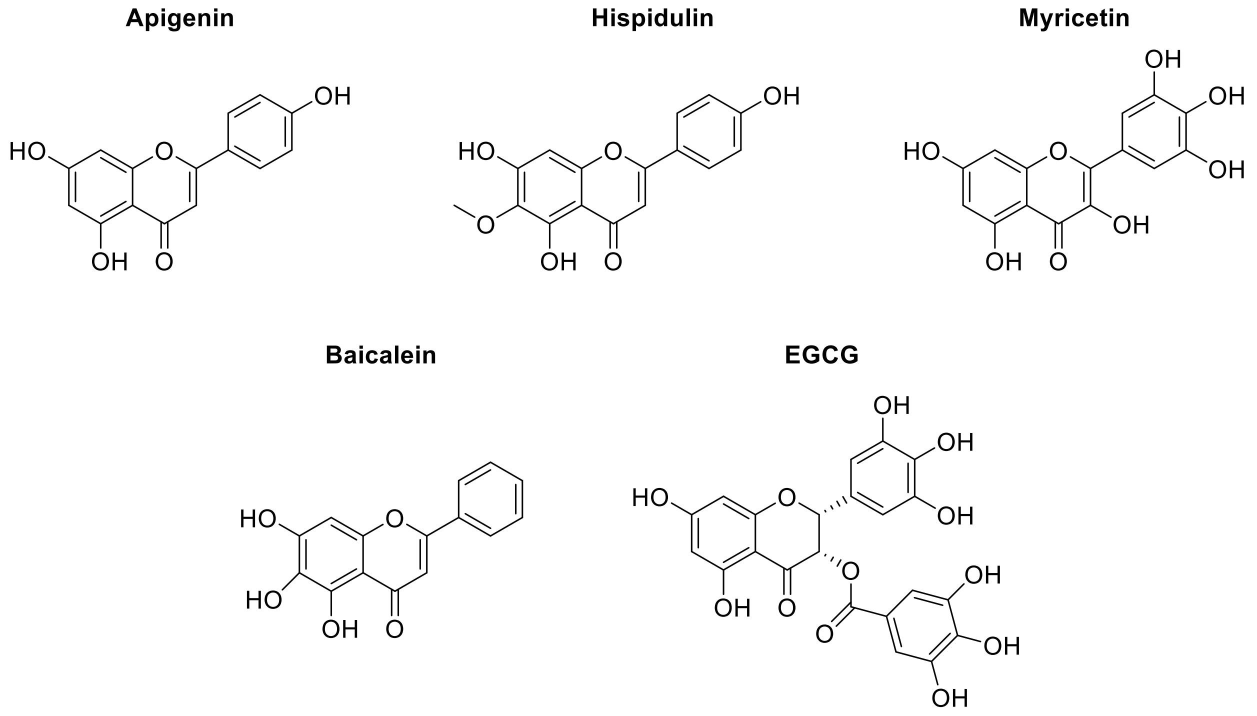

Figure S1. Chemical structures of the studied polyphenols. Polyphenols investigated for their effects on melittin, including apigenin (Api), hispidulin (Hisp), myricetin (Myr), baicalein (Bai), and epigallocatechin-3-gallate (EGCG).

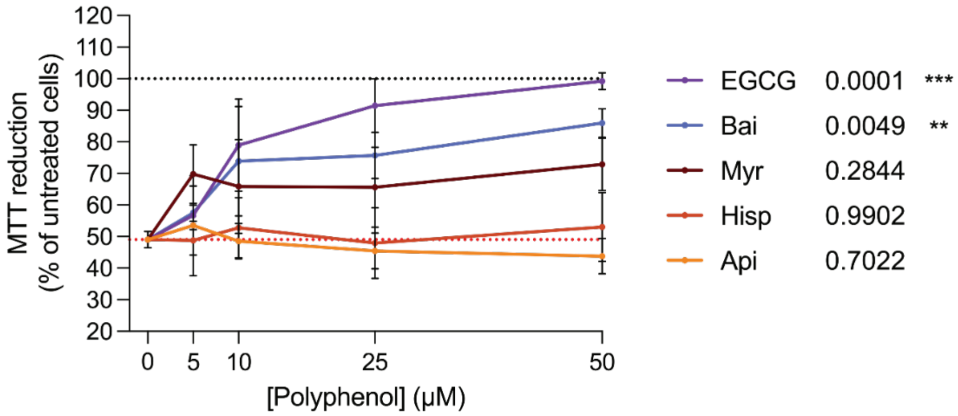

Figure S2. Additional comparison of the neutralizing effects of the five tested polyphenols against melittin toxicity. Data were analyzed for treatment effect by one-way ANOVA, with the indicated *P* values shown. Error bars indicate s.e.m. of 2-7 biologically independent experiments.

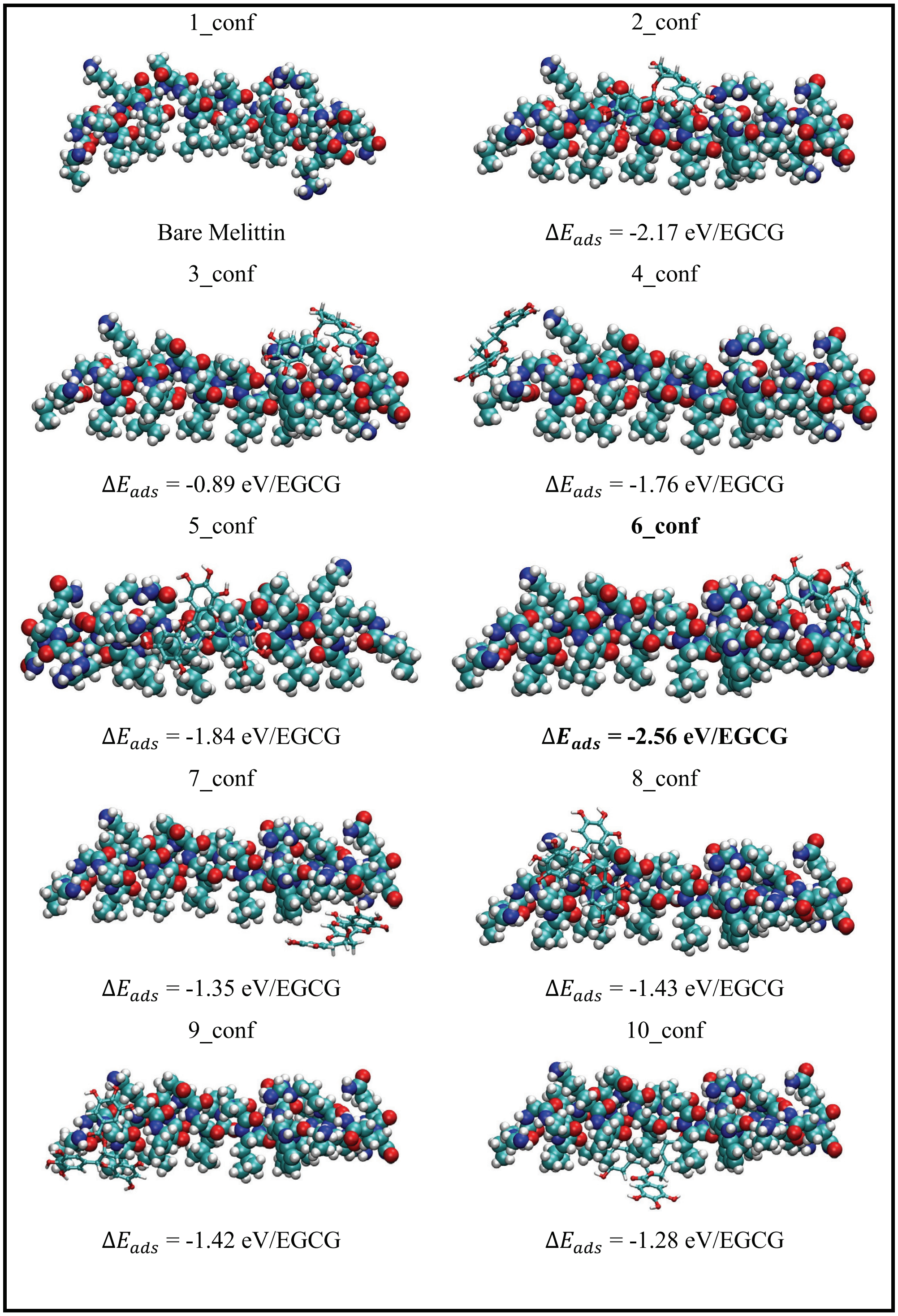

Figure S3. DFT-optimized structures of a single EGCG molecule adsorbed on the different sites of melittin, along with the corresponding ΔE_{ads} (without AIMD simulations). Among the adsorbed configurations, 6_conf (highlighted in bold) showed the highest $\Delta \boldsymbol{E}_{ads}$. Carbon, oxygen, nitrogen, and hydrogen atoms are represented by green, red, blue, and white balls, respectively. The EGCG molecule is represented using a ball-and-stick model.

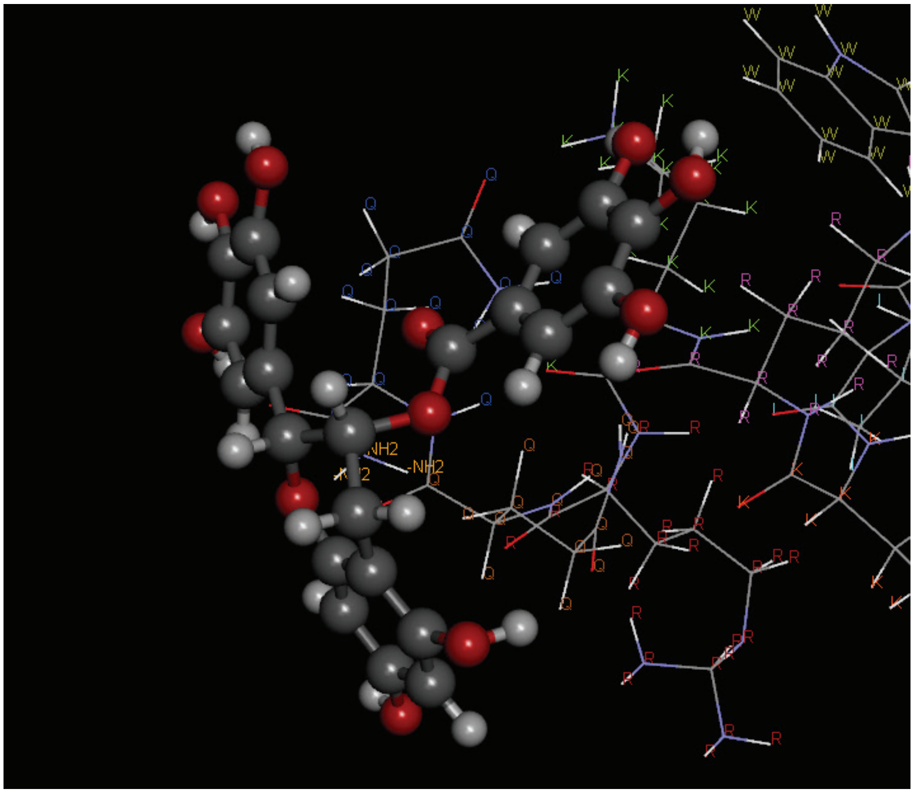

Figure S4. Interaction EGCG and melittin highlighting adsorption energy trends. Density functional theory (DFT) calculations and *ab initio* molecular dynamics (AIMD) simulations reveal that the three hydroxyl groups on the C3 benzoic ring of EGCG interact strongly with the hydrophilic amino acid residues arginine (R24 pink, R23 red), lysine (K22 green), and glutamine (26Q brown, 25Q purple) at the C-terminal end of melittin through electrostatic interactions and hydrogen bonding. These interactions are facilitated by a conformational rotation at C2 of EGCG, enabling the trihydroxyphenyl group of this polyphenol to interact favorably with the polar amino acids within the same region.

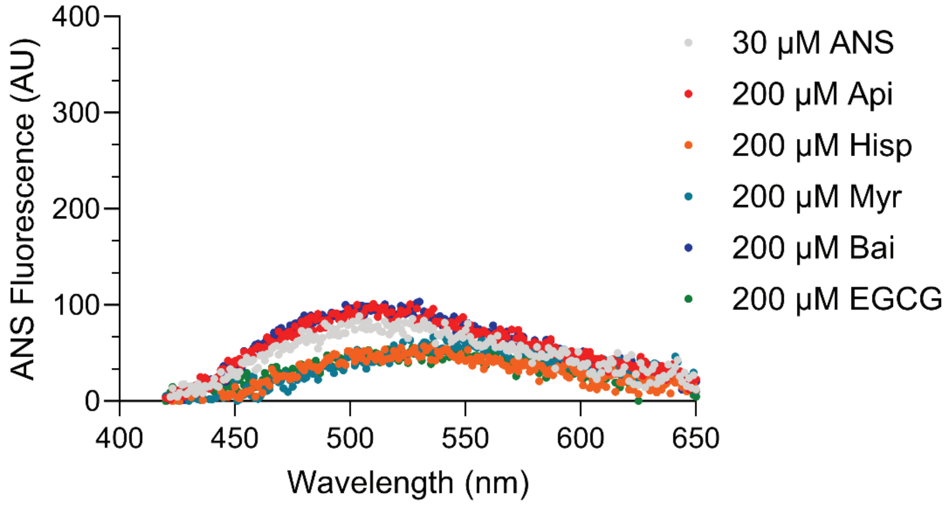

Figure S5. ANS fluorescence curves of polyphenols alone. All samples contained 30 µM 8-anilinonaphthalene-1-sulfonate (ANS), 2% dimethyl sulfoxide (DMSO), and 200 µM of the indicated polyphenol, in this case in the absence of melittin.

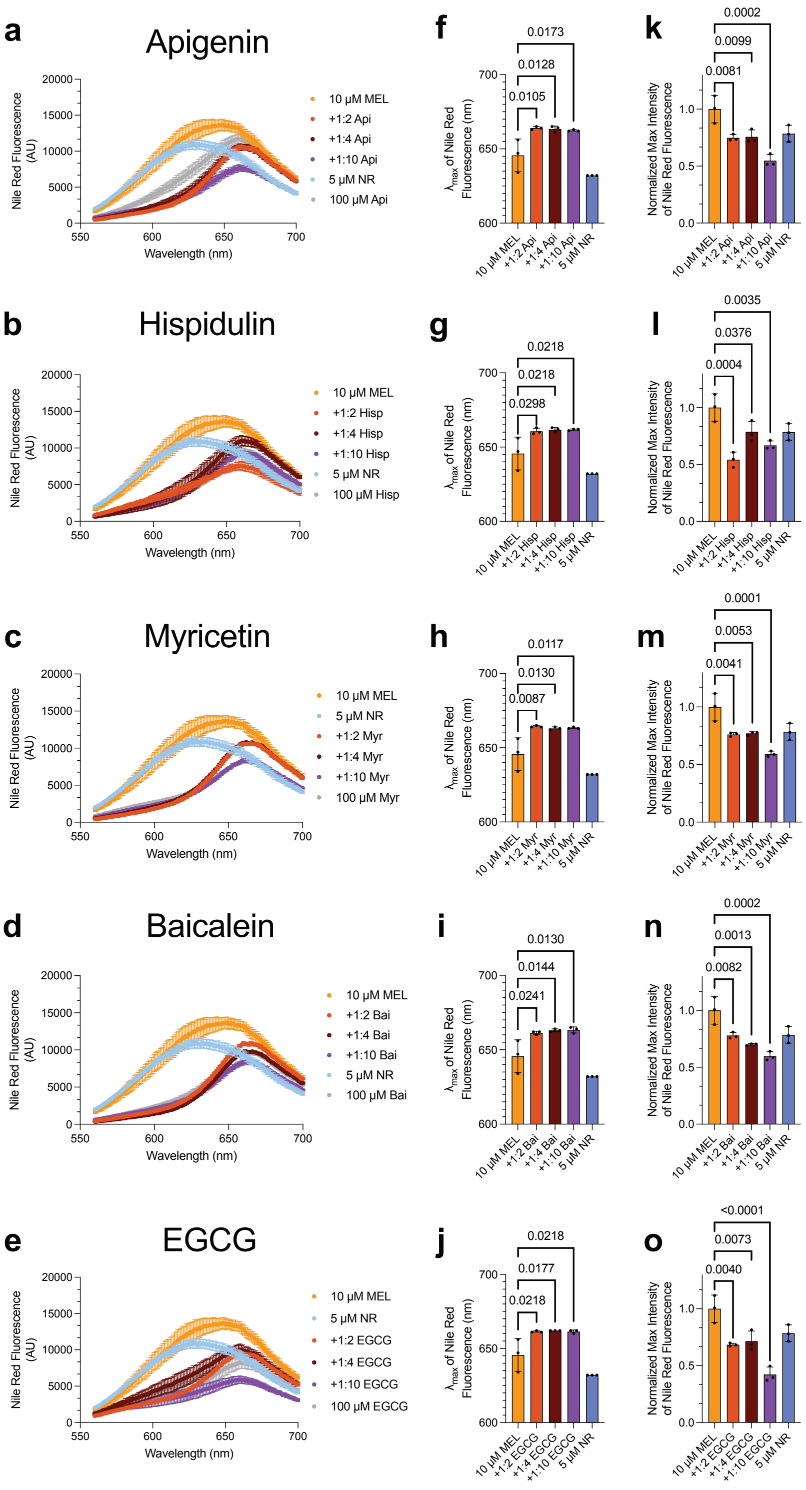

Figure S6. Nile red fluorescence measurements of melittin-polyphenol complexes. (a-e) 10 µM melittin (MEL) was incubated for 1 h at room temperature with increasing concentrations of select polyphenols (melittin-to-polyphenol ratios of 1:2 to 1:10), after which time 5 µM Nile red

(NR) was added to probe the solvent-exposed hydrophobicity of the melittin-polyphenol complexes. Free NR is shown for reference (blue). (**f-j**) Wavelength of maximum fluorescence (λ_{max}) from (a-e). (**k-o**) Normalized maximum absorbance intensity from (a-e). Samples containing melittin and select polyphenols were analyzed by one-way ANOVA followed by Dunnett's multiple comparison test. Error bars indicate the s.e.m. of three technical replicates. Data shown are representative of three independent experiments.

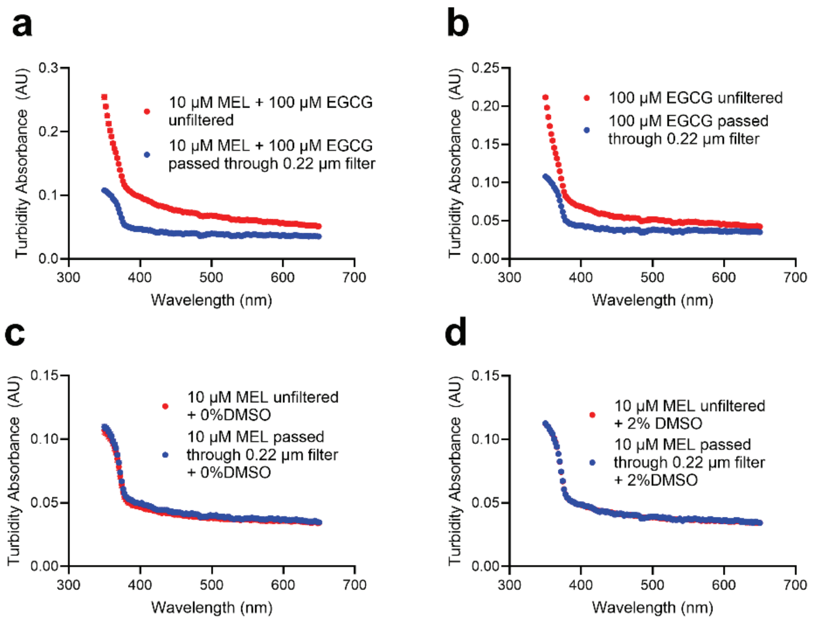

Figure S7. Size comparisons before and after filtration. Turbidity absorbance assessed with and without being passed through a 0.22 µm filter for solutions containing (**a**) 100 µM EGCG incubated with 10 µM melittin, (**b**) 100 µM EGCG alone in 2% DMSO, and (**c**) 10 µM melittin in the absence and (**d**) presence of 2% DMSO. All samples were prepared and incubated for 1 h at room temperature, as described in the Methods.

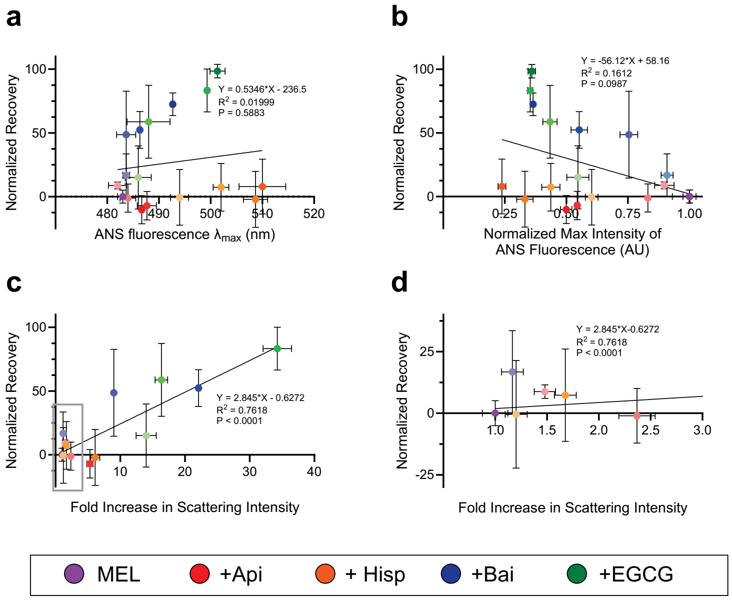

Figure S8. Expanded relationships between normalized recovery of cell health and the biophysical characteristics of melittin-polyphenol complexes from Figure 6. Correlation of the measured biophysical properties for melittin in the absence and presence of various concentrations of polyphenols. (**a**) Normalized recovery versus ANS λ_{max} for 1:2 to 1:20 molar ratios of melittin-to-polyphenol (shown in darkening colors) for Api (red), Hisp (orange), Bai (blue), and EGCG (green). (**b**) Normalized recovery versus normalized maximum ANS fluorescence intensity for the same conditions. (**c**) Normalized recovery versus the fold increase in static light scattering for the same conditions, not accounting for the background signal for the polyphenols alone. (**d**) Zoomed in version of panel c, as indicated by the grey box. Error bars indicate s.e.m. of three technical replicates.

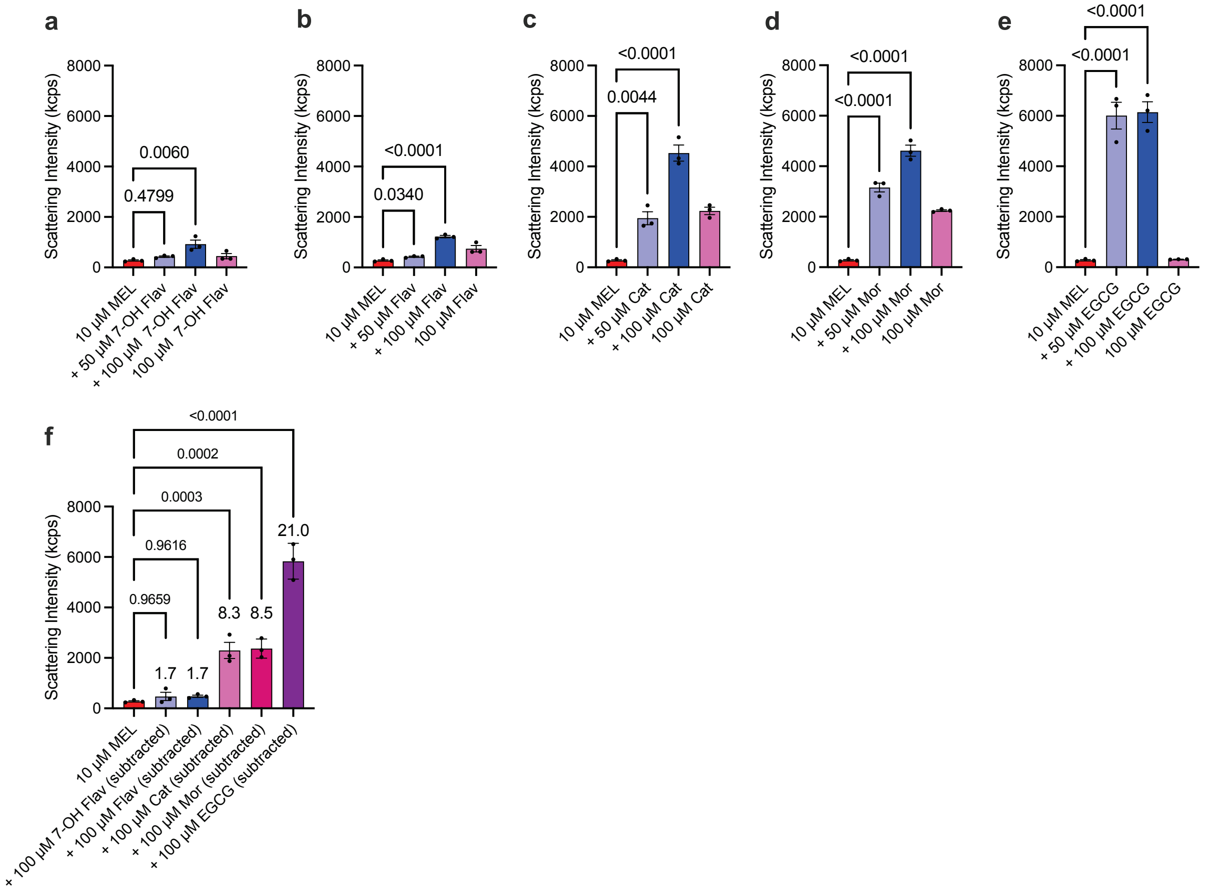

Figure S9. Additional polyphenols screened for size using static light scattering. Flavone (Flav), 7-hydroxyflavone (7-OH Flav), catechin (Cat), Morin (Mor), and EGCG were screened and showed differential increases in the sizes of the formed melittin-polyphenol complexes. Flavone has no hydroxylation, while morin and catechin are structurally similar with two disubstituted phenol rings. EGCG, with the greatest extent of hydroxylation, continued to have the largest formed melittin-polyphenol complexes. (**a-e**) 10 µM melittin (red) was incubated for 1 h at room temperature with increasing concentrations of each polyphenol (MEL-to-polyphenol ratios of 1:5 and 1:10, blue colors). 100 µM of each polyphenol was tested for reference (pink). (**f**) Conditions containing a 1:10 molar ratio of melittin-to-polyphenol were subtracted against the background signal for 100 µM polyphenol. Samples containing melittin and polyphenols were analyzed by one-way ANOVA followed by Dunnett's multiple comparison test. Error bars indicate the s.e.m. of three technical replicates. Numbers above each bar indicate the fold increase in light scattering signal relative to melittin alone.

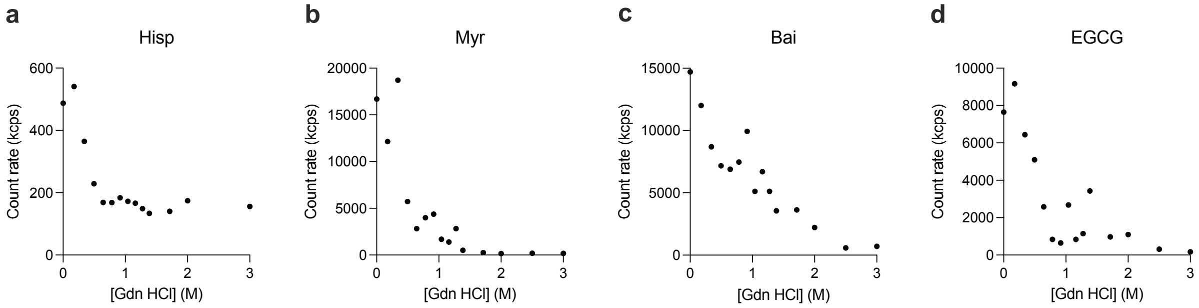

Figure S10. Raw data for guanidine hydrochloride (Gdn HCl) dissociation experiments on the stabilized melittin-polyphenol complexes. The polyphenols that attenuated the cytotoxicity of melittin were incubated for 1 h in the presence of a 10-fold molar excess of Hisp (a), Myr (b), Bai (c), or EGCG (d) and measured using static light scattering. The resulting count rate was monitored as the chaotropic agent guanidine hydrochloride (Gdn HCl) was titrated in from 0 up to 3.0 M Gdn HCl. Normalized data are shown in Figure 7.

Table S1. Cartesian coordinates (in Å) of DFT-optimized structure of melittin interacting with single EGCG molecule. Conf_6 shown in **Figure S3** was used, computationally annealed from 300 K AIMD simulation (a = 50.00 Å, b = 50.00 Å, c = 30.00 Å with α = β = γ = 90°).

N	43.9023534831	-6.7204136659	31.8313657115
C	42.5286142284	-6.5524684736	31.3320184401
C	42.4116173524	-6.8168329451	29.8294377361
O	41.3625916277	-6.5608367276	29.2016128149
N	43.5108926211	-7.3663675270	29.2651187199
C	43.6719119778	-7.5055549068	27.8190218644
C	42.5436407999	-8.3225871833	27.1833760804
O	42.0817603675	-8.0015294950	26.0786317067
C	45.0706952573	-8.0857451414	27.4846041422
C	46.2044493787	-7.1871081782	28.0374936201
C	45.2220160534	-8.3253492850	25.9736164529
C	46.1972724516	-5.7355953135	27.5386838187
N	42.0913211583	-9.4198994081	27.8534818428
C	40.9945250905	-10.2005901182	27.2989416882
C	39.6800509423	-9.4249178103	27.1349244999
O	38.8823716757	-9.7584481760	26.2409452681
N	39.4287237660	-8.4314408951	28.0219035658
C	38.2456348917	-7.5806602430	27.8694679992
C	38.3914330716	-6.6027303338	26.6884395549
O	37.4379040202	-6.3983059219	25.9203286612
C	37.9366416638	-6.8411116447	29.1749008954
N	39.5968155972	-5.9973062819	26.5508312130
C	39.9007062892	-5.1717795479	25.3784842889
C	39.6307324346	-5.9553054245	24.0774641227
O	39.0408625580	-5.4394345311	23.1203838190
C	41.3532106548	-4.6434341284	25.4477950817
C	41.6974398015	-3.8435344045	24.1817046857
C	41.5749284784	-3.7895360492	26.7080831176
N	40.0836757569	-7.2391191037	24.0543444818
C	39.8391239538	-8.0755081368	22.8852089472
C	38.3372653437	-8.2816037901	22.6481933106
O	37.8427599106	-8.0770587345	21.5206661773
C	40.5589072543	-9.4259533118	23.0499792756
C	40.2551154723	-10.4620893843	21.9511250979
C	40.6480073820	-9.9670153752	20.5505720728
C	40.9582499033	-11.7861491119	22.2904037349
N	37.5980726894	-8.7340324649	23.6862565841
C	36.2266246806	-9.1886248024	23.4638736932
C	35.2496718090	-8.0548253583	23.1641450479
O	34.2349295622	-8.3164890772	22.4757566998
C	35.7197257694	-10.0764351477	24.6112682468
C	36.4755079254	-11.4096769097	24.6996466105

C	35.9645860925	-12.3167493819	25.8339063049
C	37.0125042922	-13.3546100390	26.2843389294
N	38.1575466856	-12.7992247555	27.0353567973
N	35.5093303689	-6.8064698240	23.6072348380
C	34.6463879101	-5.7074617623	23.1640087512
C	34.7421862181	-5.4909356472	21.6394173596
O	33.8387955557	-4.8823055775	21.0420663501
C	34.8560059711	-4.3863548699	23.9359332463
C	34.5491655020	-4.5654610159	25.4313577925
C	36.2363461494	-3.7589759538	23.7014214404
N	35.8413267865	-5.9984069564	21.0295624470
C	35.9631005069	-6.0637681839	19.5802401061
C	35.3591289827	-7.3763214093	19.0361952520
O	34.4027694057	-7.3612357383	18.2373312696
C	37.4211460052	-5.8605132870	19.1273815876
C	38.1102376787	-4.6300636242	19.7507843443
C	39.5670203074	-4.5568929717	19.2658528765
C	37.3613383784	-3.3216869170	19.4607092600
N	35.9279369219	-8.5401551650	19.4557253727
C	35.5657783742	-9.8054657387	18.8576478055
C	34.0863750682	-10.2430043110	19.0509858477
O	33.5156553454	-10.8812478586	18.1601049222
C	36.4422897423	-10.9373185143	19.4145624250
O	37.0386260238	-10.8017268286	20.4779530934
C	36.4934512578	-12.1934567824	18.5884405075
N	33.5394343355	-9.9201377345	20.2481902627
C	32.1385228700	-10.1462174915	20.5158155686
C	31.2825451342	-8.8620821551	20.3869895594
O	30.1646442672	-8.8813196233	19.8419475913
C	31.9252890889	-10.8312240650	21.8887310684
O	32.8788881768	-11.2004572899	22.5578650222
C	30.4914907430	-11.0031649507	22.3253474670
N	31.8360990038	-7.7411202471	20.9110019312
C	31.0926814120	-6.4892396020	20.9219302699
C	30.7306914751	-5.9461555536	19.5402083557
O	29.6052521171	-5.4410598904	19.3573729760
N	31.6851555178	-5.9968417552	18.5804419481
C	31.4236528082	-5.4159142840	17.2676644843
C	30.4512196716	-6.2580239070	16.4352700838
O	29.5205026227	-5.6788796482	15.8386321663
C	32.7111454735	-5.0608634497	16.5125047571
C	33.5176753936	-3.9156214431	17.1586115245
C	34.8669555871	-3.7781063277	16.4363708639
C	32.7505864930	-2.5844816636	17.1483996403
N	30.5618827375	-7.6083058295	16.3991041499
C	29.4899698144	-8.3803548252	15.7397341793

C	28.1093595464	-8.1004409295	16.3377534996
O	27.1147743595	-7.9991301093	15.5972572351
C	29.9649990362	-9.8399962214	15.8872472697
C	31.4961453097	-9.7063974716	15.8906450725
C	31.7226631601	-8.4588431966	16.7511234601
N	28.0307062640	-7.9997980326	17.6874521333
C	26.7613519869	-7.6663645530	18.3350782685
C	26.2336867418	-6.2904373736	17.8804757044
O	25.0446855122	-6.1612604868	17.5243547913
C	26.9100775904	-7.7271382634	19.8582408471
N	27.1113355097	-5.2660435506	17.9133950075
C	26.7229027472	-3.9191387819	17.4938299620
C	26.2771272240	-3.8741323985	16.0229791806
O	25.2836318201	-3.2026341897	15.6830414804
C	27.8927900353	-2.9503047014	17.7462691822
C	27.6432361969	-1.4992740233	17.2967322223
C	26.4360828509	-0.8622586086	18.0026969428
C	28.9174649359	-0.6689962209	17.5224604482
N	27.0356357391	-4.5572361973	15.1385224070
C	26.6598970757	-4.6268835881	13.7294996289
C	25.2655915075	-5.2506009447	13.5787016783
O	24.3961929688	-4.7158012033	12.8722477403
C	27.7467487933	-5.3773137935	12.9236849744
C	29.0422042879	-4.5373487186	12.8735055542
C	27.2403141645	-5.7270152235	11.5166763751
C	30.2793594702	-5.3508876171	12.4705807206
N	25.0411995157	-6.4081052272	14.2486998016
C	23.7252295821	-7.0230671488	14.2034942241
C	22.5991705388	-6.0648750332	14.6968636568
O	21.5239626706	-5.9973725395	14.0833451338
C	23.6861314445	-8.2544781253	15.1037875070
O	22.9581647607	-9.2051571570	14.8798238182
N	22.8893937545	-5.3881467953	15.8280382672
C	21.9416262938	-4.4592391843	16.4438787332
C	21.5729050651	-3.3120647296	15.4862594372
O	20.3890861975	-2.9542993374	15.3570245008
C	22.5460947126	-3.9560028017	17.7727452987
C	21.5531905455	-3.4189516803	18.7597724940
C	20.2966874612	-2.8865281221	18.5377113180
C	21.7368849083	-3.4396044939	20.1899151204
N	19.6945123366	-2.5610450315	19.7438935275
C	20.5409711881	-2.9227737499	20.7752907546
C	22.7769943616	-3.8814021548	21.0308577951
C	20.3587310349	-2.8627886829	22.1625234945
C	22.5992440958	-3.8149506639	22.4109953080
C	21.4019463054	-3.3154390865	22.9702048184

N	22.5924429465	-2.7350094790	14.8080614480
C	22.3422971516	-1.7110649859	13.7959138699
C	21.4502815350	-2.2551094293	12.6691513256
O	20.4820687167	-1.5961869117	12.2403037999
C	23.6850710424	-1.1498860273	13.2671663589
C	24.3944624294	-0.3399940363	14.3745229826
C	23.4690272648	-0.3108497388	11.9981145602
C	25.8753443871	-0.0693572575	14.0775692898
N	21.7677093741	-3.4683999689	12.1661512086
C	20.9477013583	-4.0476269387	11.1034084557
C	19.4750387013	-4.1709076857	11.5243034200
O	18.5549029632	-3.9239282212	10.7174005834
C	21.5145109305	-5.3937705950	10.6306395882
C	22.8739785213	-5.2495147198	9.9318121047
C	23.4139052040	-6.5885344919	9.4127671862
C	24.7272988839	-6.4343764149	8.6287315635
N	25.3591715436	-7.6802180285	8.1611118206
N	19.2276250574	-4.5528676820	12.8001512668
C	17.8639345620	-4.6510153720	13.3044165739
C	17.0913737426	-3.3263808577	13.2475062629
O	15.8408728769	-3.3588518129	13.1995452689
C	17.8506524177	-5.1705433981	14.7515372730
C	18.3319565846	-6.6216079644	14.8794124911
C	18.2098085977	-7.1622982117	16.3077894449
N	18.8738587283	-6.2733943204	17.2783746372
C	18.9602475524	-6.8433358370	18.6278959788
N	20.0842137627	-7.7581054372	18.8004256822
N	19.0419476965	-5.6914342852	19.5406294762
N	17.8030094394	-2.1804389586	13.2864835621
C	17.1719576266	-0.8669500586	13.2354722322
C	16.5550637197	-0.5306773736	11.8692246669
O	15.8343529198	0.4819446127	11.7592666280
C	18.1672919743	0.2469671826	13.6144710217
C	18.7764787589	0.1455141118	15.0199427469
C	17.7511786012	0.0828828186	16.1623524625
C	18.3886848613	0.4346833358	17.5083964942
N	17.4417663642	0.2439629732	18.6379953781
N	16.8031956278	-1.3710674706	10.8354291318
C	16.0895972426	-1.2591985313	9.5675609535
C	14.6589651399	-1.8640554381	9.5952804413
O	13.9992122080	-1.9196871532	8.5562083104
C	16.9019107021	-1.8039095772	8.3871027338
C	18.1257712029	-0.9451463213	7.9995360525
C	19.4933466658	-1.4371085019	8.5346648828
N	19.9082881039	-2.8032706850	8.2115089264
C	20.0640480756	-3.1791622361	6.8127002623

N	20.8813105781	-4.4004097760	6.8003118702
N	18.8805047035	-3.5194984688	6.0018252834
N	14.1762937815	-2.2313390949	10.8215401205
C	12.7442700739	-2.3436823799	11.0936223086
C	12.1395181094	-0.9320674794	11.2347225675
O	11.5962838815	-0.3984980288	10.2464464851
C	12.5059714475	-3.2223977684	12.3208223004
C	12.7875720065	-4.7089882936	12.0354004427
C	12.9113513981	-5.4633076741	13.3414242344
O	11.9168969203	-5.8413955806	13.9995792241
N	14.1850374600	-5.6799133770	13.7647353744
N	12.2408687792	-0.3322502081	12.4497759536
C	11.9123815018	1.0755864519	12.7331021283
C	10.3897177237	1.3527970659	12.7411577708
O	9.8409206381	1.9931774897	13.6721754517
C	12.7000006060	2.0609167458	11.8216678945
C	13.0561028123	3.3840736408	12.5174297728
C	13.9977442860	3.3184116869	13.7153118236
O	13.9963319332	4.2343976616	14.5663372648
N	14.8040867045	2.2358466359	13.8345645229
N	9.6957656781	0.8867772123	11.6893057557
H	44.3675859097	-5.8202774016	31.9467689582
H	43.9180188520	-7.1840393362	32.7382068327
H	41.8526387078	-7.2626027769	31.8296557411
H	42.1001863212	-5.5542041067	31.5060469479
H	44.3078362253	-7.4493816017	29.9059233156
H	43.5723957148	-6.5163568359	27.3481889537
H	45.1453852320	-9.0601146864	27.9940848304
H	46.1932238429	-7.1939899123	29.1381590526
H	47.1554976515	-7.6668190440	27.7631809185
H	46.2374616840	-8.6782347772	25.7512431734
H	45.0477665902	-7.4079724588	25.3968713429
H	44.5146791712	-9.0775737153	25.6025994806
H	46.2377627879	-5.6781007229	26.4433397842
H	47.0684818729	-5.1899102531	27.9265524164
H	45.3025098217	-5.1887770281	27.8663706158
H	42.4599096359	-9.6246390285	28.7771476827
H	41.2562418564	-10.5661658265	26.2978053730
H	40.8133311041	-11.0742049727	27.9392738236
H	40.1920789517	-8.0674694879	28.5992153552
H	37.4083981797	-8.2298530379	27.5861872956
H	38.7862940854	-6.2207434438	29.4881415671
H	37.7097709250	-7.5563995565	29.9758311404
H	37.0631306889	-6.1912674399	29.0323985647
H	40.3520440222	-6.2365631832	27.1924536755
H	39.2018346060	-4.3239233944	25.3445033230

H	42.0115434635	-5.5245959307	25.4951455292
H	42.7272914891	-3.4677320371	24.2385422357
H	41.0302098312	-2.9779146870	24.0683937626
H	41.6050998769	-4.4483234613	23.2703208146
H	42.6105546965	-3.4230180185	26.7394680985
H	41.3915614521	-4.3443264576	27.6381131682
H	40.9140198659	-2.9113100268	26.7056761686
H	40.6426741165	-7.6095395914	24.8257356582
H	40.1860381058	-7.5479140875	21.9870894718
H	41.6416007879	-9.2310707949	23.0930733833
H	40.2738489683	-9.8494235401	24.0255419782
H	39.1713503011	-10.6484882874	21.9445259260
H	40.4555750064	-10.7456551852	19.7996567427
H	41.7168322299	-9.7157912881	20.5025917415
H	40.0709288504	-9.0794993845	20.2572472834
H	40.7224145733	-12.5602177740	21.5474135564
H	40.6508582677	-12.1657714353	23.2756570504
H	42.0507833672	-11.6651004631	22.3041948594
H	38.0386592691	-8.9027844744	24.5952070975
H	36.2247276992	-9.7832427022	22.5381395949
H	35.7995089606	-9.5272739860	25.5617669152
H	34.6509559109	-10.2615669815	24.4283096959
H	36.3937024955	-11.9357831650	23.7370558848
H	37.5441573383	-11.2060951606	24.8427582827
H	35.6900192437	-11.7044417516	26.7064403513
H	35.0389843302	-12.8204429350	25.5146511074
H	36.5401580209	-14.1205034273	26.9139642965
H	37.4083318124	-13.8861914568	25.4071896407
H	37.8061431728	-12.4149841959	27.9150329030
H	38.5241511049	-11.9908245586	26.5259518003
H	36.3161960031	-6.6351415061	24.2162319245
H	33.6054053724	-6.0237790783	23.3238837586
H	34.0974702250	-3.7162328468	23.5028894149
H	34.5689316992	-3.5925640295	25.9410919303
H	35.2912927047	-5.2100657135	25.9202659142
H	33.5552376819	-5.0066451520	25.5889009072
H	36.3042417833	-2.7977221471	24.2284149951
H	36.4232152413	-3.5704780689	22.6355020479
H	37.0505283073	-4.3973253707	24.0654864642
H	36.5356498502	-6.5125606335	21.5736147460
H	35.3204830437	-5.2745795930	19.1750896386
H	38.0090926493	-6.7529011409	19.3820273672
H	37.4216208745	-5.7782541013	18.0293063029
H	38.1346820685	-4.7807395778	20.8389680560
H	40.0981656699	-3.7203517120	19.7404473477
H	39.6222519784	-4.4098982996	18.1779204701

H	40.1143364621	-5.4791922040	19.5081037473
H	37.8756102949	-2.4688830473	19.9248910036
H	36.3332168733	-3.3365594822	19.8496534269
H	37.3076080554	-3.1274322427	18.3800332425
H	36.6512191597	-8.5420004200	20.1921845061
H	35.6722324391	-9.7593570161	17.7641758370
H	37.1580905947	-12.0213096438	17.7263304630
H	35.5005373613	-12.4314354797	18.1786842819
H	36.8883737597	-13.0220165504	19.1852844795
H	34.0926383475	-9.4621122354	20.9868252324
H	31.7542764787	-10.8276106890	19.7404092625
H	29.8621906226	-11.3682713611	21.5015454749
H	30.0795314281	-10.0230765128	22.6149623787
H	30.4452679264	-11.6808439560	23.1839321071
H	32.7240347915	-7.7918104928	21.4250490330
H	30.1429498753	-6.6027221829	21.4588111819
H	31.7196821234	-5.7362522685	21.4204095166
H	32.6055753407	-6.4072558252	18.7671188594
H	30.8447062879	-4.4986804319	17.4352026481
H	33.3535136241	-5.9478482369	16.4329751355
H	32.4252259834	-4.7732717517	15.4888321957
H	33.7096880114	-4.1860193526	18.2080185542
H	35.4747867618	-2.9772117867	16.8780525190
H	34.7232864702	-3.5364244136	15.3736892183
H	35.4459641941	-4.7113205302	16.4914698158
H	33.3595869553	-1.7809422058	17.5848627735
H	31.8190039093	-2.6334159506	17.7292051199
H	32.4910441858	-2.2859801313	16.1226624252
H	29.4114969469	-8.0864026193	14.6835891424
H	29.6213456707	-10.2486378751	16.8473917895
H	29.5700309602	-10.4738415376	15.0847778824
H	32.0135057203	-10.5758904142	16.3120444502
H	31.8668408409	-9.5364663217	14.8711271825
H	32.6672771819	-7.9486891053	16.5477931489
H	31.6970349955	-8.7135272440	17.8153959341
H	28.8658218911	-8.0993415068	18.2697562655
H	25.9967984583	-8.3767374597	17.9936877364
H	25.9504416256	-7.4859038198	20.3331330228
H	27.6662282592	-7.0099760372	20.2042257218
H	27.2179157701	-8.7322836454	20.1733222580
H	28.0674314527	-5.4111038037	18.2632889502
H	25.8347942285	-3.6123396972	18.0618835844
H	28.1304271820	-2.9739371585	18.8204509530
H	28.7768905572	-3.3450909970	17.2224238731
H	27.4320440383	-1.5101624630	16.2177262541
H	26.3202623842	0.1883529307	17.7022362171

H	26.5593312446	-0.8824909851	19.0945448470
H	25.4992263678	-1.3779717021	17.7520063441
H	29.1734748644	-0.6191201739	18.5903666180
H	28.7860808430	0.3620763351	17.1668378380
H	29.7808867863	-1.0988803530	16.9942808965
H	27.8563706669	-5.0821795046	15.4649872145
H	26.5407083217	-3.6071587542	13.3335698500
H	27.9579036169	-6.3130330226	13.4654716118
H	28.8875791124	-3.7005350675	12.1766084209
H	29.2251344405	-4.0891859320	13.8586778523
H	28.0263644045	-6.2129646780	10.9251678139
H	26.9228620376	-4.8241173669	10.9767685188
H	26.3815666705	-6.4109134098	11.5477024862
H	30.1765393855	-5.8069879785	11.4773109148
H	30.4606683808	-6.1582718280	13.1939696741
H	31.1796754110	-4.7211089602	12.4475755242
H	25.7835343071	-6.8421769441	14.8119570769
H	23.4365371277	-7.3022938729	13.1820542926
H	24.3345103087	-8.1946065758	16.0038737998
H	23.7965687185	-5.5313983076	16.2892819386
H	20.9958336986	-4.9920317544	16.6320945220
H	23.0600649585	-4.8092675339	18.2408678828
H	23.3403902259	-3.2242908292	17.5502070817
H	19.7741974879	-2.7398085007	17.5989796973
H	18.6724133920	-2.4580038928	19.8068830042
H	23.7002375652	-4.2742640711	20.6078002450
H	19.4349580890	-2.4804854190	22.5911086038
H	23.3916719416	-4.1553034306	23.0738405582
H	21.2914066442	-3.2842739483	24.0517285450
H	23.5542271796	-3.0600239563	14.9587907958
H	21.7508043643	-0.8973640605	14.2402102329
H	24.3128964598	-2.0184230707	13.0116184281
H	23.8526188026	0.6066366336	14.5167338164
H	24.3244488517	-0.8822855533	15.3263908527
H	23.0599252005	-0.9096853238	11.1735952166
H	24.4162687944	0.1228666911	11.6539361028
H	22.7671555860	0.5136039292	12.1845699817
H	26.4238187726	-1.0153389634	13.9622064999
H	26.3501110806	0.4872968306	14.8979835816
H	26.0208689363	0.5146551199	13.1597872297
H	22.5559452565	-3.9989764224	12.5478106715
H	20.9302662788	-3.3546491968	10.2491416674
H	21.5857750226	-6.0816397125	11.4862855507
H	20.7882759762	-5.8243875857	9.9252537763
H	22.7682105530	-4.5377088110	9.0991098283
H	23.6081994993	-4.8073510232	10.6186603233

H	23.5778051585	-7.2726688712	10.2609448370
H	22.6620488268	-7.0750454533	8.7720738397
H	24.5566782652	-5.7953509047	7.7502796828
H	25.4618331940	-5.8989000347	9.2467657486
H	24.7076987426	-8.1923888578	7.5635803268
H	25.5394996562	-8.2917653310	8.9593999239
H	20.0109772960	-4.7677655036	13.4233551192
H	17.2945816692	-5.3293365999	12.6512125382
H	18.4868458780	-4.4993626732	15.3472186447
H	16.8251215458	-5.0679457191	15.1386612810
H	17.7421158780	-7.2653828805	14.2090363219
H	19.3791322604	-6.6952391273	14.5600285026
H	17.1471270218	-7.3173692422	16.5679990967
H	18.7012352436	-8.1449758409	16.3564369805
H	18.3368296632	-5.4044442149	17.3338268007
H	18.0530250702	-7.4092453479	18.9005937838
H	20.8992791829	-7.3823568372	18.3076819665
H	19.8774054358	-8.6653711142	18.3811901655
H	19.2937647430	-6.0257809672	20.4739321531
H	19.8088223549	-5.0779922372	19.2351532888
H	18.8240653618	-2.2178874369	13.2020918203
H	16.3149152025	-0.8790654121	13.9286104535
H	18.9803919212	0.2485845632	12.8744339227
H	17.6270540764	1.1971009435	13.5021363991
H	19.4336106084	-0.7339017183	15.0833300974
H	19.4276152842	1.0233932256	15.1552316771
H	16.9113199479	0.7709106183	15.9708724441
H	17.3099846443	-0.9220709220	16.2085416631
H	19.2581845738	-0.2126992953	17.6914912042
H	18.7620616164	1.4693338432	17.4718983090
H	16.5381999392	0.7041669605	18.4648484085
H	17.8188627237	0.6621111834	19.4915078814
H	17.3957889812	-2.1958935402	10.9770544617
H	15.8953678691	-0.1864878747	9.4116275454
H	17.2070609688	-2.8369852338	8.6069872136
H	16.1922025397	-1.8660621022	7.5512971777
H	18.1831989943	-0.8629213261	6.9043860456
H	17.9780082200	0.0872689155	8.3534555234
H	20.2725071074	-0.7512149248	8.1688887635
H	19.5106484489	-1.3597845798	9.6313563827
H	19.3496653706	-3.4885830040	8.7279028402
H	20.5368412652	-2.3151273484	6.3154808101
H	21.7731761457	-4.2174895097	7.2598496115
H	21.0588572174	-4.6780297284	5.8342899685
H	18.2272550065	-2.7373213652	5.9811868692
H	18.3970705874	-4.3040752386	6.4456523421

H	14.7982576572	-2.2185342056	11.6304879924
H	12.2674774600	-2.7593339861	10.1963227703
H	13.1573325265	-2.8814715405	13.1368887284
H	11.4739393226	-3.0935978414	12.6670882729
H	11.9605475856	-5.1435242350	11.4586326336
H	13.7124523230	-4.8069858966	11.4527768445
H	14.9087591593	-5.0143597251	13.4497252334
H	14.2849436927	-6.0310860156	14.7139817630
H	12.7054250517	-0.8192582772	13.2213235739
H	12.2401650310	1.2208388345	13.7679775151
H	13.6084856387	1.5434924166	11.4849290661
H	12.1247770782	2.2674894469	10.9102463894
H	13.5193831012	4.0678973759	11.7878498962
H	12.1601956148	3.9001296275	12.8893812887
H	15.0024572638	1.5589283849	13.0847926332
H	15.4250178409	2.2251131121	14.6376400543
H	10.2054481554	0.3766097953	10.9469958124
H	8.6810998424	0.8847015341	11.7366537469
O	9.3572439250	-1.4383102475	16.9509585778
O	12.2484367500	-2.3164523194	15.5906989080
O	9.5255148233	-4.6367549545	13.2866071885
O	7.0435334955	-0.6414991031	12.8052465840
O	11.4458071493	2.8844241806	15.5224753514
O	14.5570888198	1.2763241292	18.7200121873
O	14.0731536265	-1.2033112265	14.9590092494
O	13.8395687579	3.1295241950	17.0335979229
O	14.7982458715	-6.2555589856	17.9172667669
O	16.9592622407	-2.2371176565	19.2780652852
O	16.4825506200	-5.0425408107	19.5269676163
C	11.4471434450	-2.8007570636	16.7134859245
C	10.7241155788	-1.6241758811	17.4208439302
C	10.4435175600	-3.8004304962	16.1295838899
C	9.5327668752	-3.0640036203	15.1814508652
C	11.5010007394	-0.3182735865	17.3655678453
C	9.0491573785	-1.8362778981	15.6595000416
C	9.1552393047	-3.4583679181	13.8795452413
C	8.2109012961	-1.0062959222	14.9104523968
C	12.6490859012	-0.1367874925	18.1572994272
C	11.1012079761	0.6797658561	16.4760249437
C	8.3152406864	-2.6343515312	13.1071265066
C	7.8525615291	-1.4266147224	13.6259696556
C	13.5827719981	-2.0339087972	15.7227627731
C	11.8268416307	1.8732100272	16.3445056696
C	13.3916739821	1.0351360844	17.9926765452
C	13.0109616423	2.0328282590	17.0850956119
C	14.3764384285	-2.8245064516	16.6995723195

C	15.3240366309	-2.1646748980	17.4890992769
C	14.1787476634	-4.2088662688	16.8310795012
C	14.9516000445	-4.9028514874	17.7566108944
C	16.0810637847	-2.8620531289	18.4468924784
C	15.8965155883	-4.2529884040	18.5764531245
H	12.1078368356	-3.2762989477	17.4455038646
H	10.5979181001	-1.9289175014	18.4709997236
H	9.8653492196	-4.2264462482	16.9661324851
H	10.9845340384	-4.6249744477	15.6506930746
H	7.8863436285	-0.0518331345	15.3170172859
H	12.9791307739	-0.8843363209	18.8738690165
H	10.2060254054	0.5536498007	15.8769675981
H	8.0388620363	-2.9467678287	12.1050809215
H	10.3414615189	-5.0500899575	13.6841190385
H	6.7538456929	0.1223041616	13.3332650466
H	15.4117265473	-1.0876421305	17.4017367001
H	13.4330629288	-4.7360465396	16.2423733182
H	10.7568754676	2.5467296749	14.8627919416
H	14.7877665227	2.1981208696	18.4674998596
H	13.7870988370	3.6047086631	16.1470519441
H	15.4058194057	-6.4746949177	18.6596110135
H	17.0966353203	-1.2274564967	18.9830810443
H	17.5179904728	-5.0645831975	19.5362338931
